# Differentiating Nanomaghemite and Nanomagnetite and Discussing Their Importance in Arsenic and Lead Removal from Contaminated Effluents: A Critical Review

**DOI:** 10.3390/nano11092310

**Published:** 2021-09-06

**Authors:** Juan A. Ramos-Guivar, Diego A. Flores-Cano, Edson Caetano Passamani

**Affiliations:** 1 Grupo de Investigación de Nanotecnología Aplicada para Biorremediación Ambiental, Energía, Biomedicina y Agricultura (NANOTECH), Facultad de Ciencias Físicas, Universidad Nacional Mayor de San Marcos, Av. Venezuela Cdra 34 S/N, Ciudad Universitaria, Lima 15081, Perú; diego.flores4@unmsm.edu.pe; 2 Physics Department, Federal University of Espírito Santo, Vitória 29075-910, ES, Brazil; passamaniec@yahoo.com.br

**Keywords:** nano-γ-Fe_2_O_3_, nano-Fe_3_O_4_, arsenic, lead, contaminated effluents, water purification

## Abstract

Arsenic and lead heavy metals are polluting agents still present in water bodies, including surface (lake, river) and underground waters; consequently, the development of new adsorbents is necessary to uptake these metals with high efficiency, quick and clean removal procedures. Magnetic nanoparticles, prepared with iron-oxides, are excellent candidates to achieve this goal due to their ecofriendly features, high catalytic response, specific surface area, and pulling magnetic response that favors an easy removal. In particular, nanomagnetite and maghemite are often found as the core and primary materials regarding magnetic nanoadsorbents. However, these phases show interesting distinct physical properties (especially in their surface magnetic properties) but are not often studied regarding correlations between the surface properties and adsorption applications, for instance. Thus, in this review, we summarize the main characteristics of the co-precipitation and thermal decomposition methods used to prepare the nano-iron-oxides, being the co-precipitation method most promising for scaling up processes. We specifically highlight the main differences between both nano-oxide species based on conventional techniques, such as X-ray diffraction, zero and in-field Mössbauer spectroscopy, X-ray photoelectron spectroscopy, X-ray absorption spectroscopy, and X-ray magnetic circular dichroism, the latter two techniques performed with synchrotron light. Therefore, we classify the most recent magnetic nanoadsorbents found in the literature for arsenic and lead removal, discussing in detail their advantages and limitations based on various physicochemical parameters, such as temperature, competitive and coexisting ion effects, i.e., considering the simultaneous adsorption removal (heavy metal–heavy metal competition and heavy metal–organic removal), initial concentration, magnetic adsorbent dose, adsorption mechanism based on pH and zeta potential, and real water adsorption experiments. We also discuss the regeneration/recycling properties, after-adsorption physicochemical properties, and the cost evaluation of these magnetic nanoadsorbents, which are important issues, but less discussed in the literature.

## 1. Introduction

Bulk magnetite (Fe_3_O_4_) and maghemite (γ-Fe_2_O_3_) are common iron-oxides (Fe-oxides) found in nature as minerals and in the corrosion industry [1,2]. However, with the appearance of nanotechnology and advances in new routes of synthesis (including physical, chemical, and biosynthesis), many researchers have tried to prepare either pure ˈnanomagnetiteˈ (nano-Fe_3_O_4_) or nanomaghemite (nano-γ-Fe_2_O_3_) or a mixture of these phases forming the core–shell-like nanosystems [3,4,5,6,7,8,9,10]. Moreover, it should be emphasized that these materials can exist in a ferrofluid form, magnetic nanopowders, solid magnetic matrices, core–shell-like structures, and magnetic colloid solutions. However, in a nanoscale regime, the results may suggest that it is hard to compare different nano-Fe_3_O_4_ or nano-γ-Fe_2_O_3_ when they are prepared from different routes. For instance, the three main issues found in nano-oxide systems are (i) the fast conversion from nano-Fe_3_O_4_ to nano-γ-Fe_2_O_3_, (ii) the different optical, thermal, magnetic, electronic, mechanical, textural, colloidal, surface, and adsorptive properties due to different Fe-oxide growth mechanisms [3,4,5,6,7,8,9,10,11], and (iii) the problem related to a critical size of the nanoparticles (NPs), i.e., below a certain critical radius (~10 nm), these two Fe-oxide phases are rather difficult to differentiate even from the perspective of different conventional techniques, such as powder X-ray diffraction (pXRD), zero-field Mössbauer spectroscopy (MS), X-ray photoelectron spectroscopy (XPS), and Vibrating Sample Magnetometry (VSM) measurements, often yielding a bad interpretation and characterization of the correct Fe-oxide phase presented in the sample. According to the literature, many scientific works agree that this critical radius is located ca. 10 nm [3,4,5,6,7,8,9,10,11]. Below this critical size, nanoparticle (NP) surface magnetic effects are huge and considerable, giving rise to coexisted competitive magnetic effects. Unfortunately, most review reports have discussed Fe-oxide-based samples with sizes superior to this critical radius; hence, some interesting effects, such as superparamagnetism and superspinglass, are not fully studied. For example, da Costa et al. [12] reported an interesting review about γ-Fe_2_O_3_ and magnetite, but the studied particle sizes were bigger than 10 nm; consequently, no superparamagnetic relaxation Mössbauer spectra were observed for temperatures below 300 K. Another relevant review focus only on γ-Fe_2_O_3_ NPs was conducted by Tuček et al. [13] more than a decade ago.

In particular, while Cornell et al. [1] summarized very well the distinct crystallographic and magnetic properties of Fe_3_O_4_ and γ-Fe_2_O_3_ at a bulk state, Greneche [14] devoted decades to the study of nano-γ-Fe_2_O_3_ and composites, including core–shell models. Regarding this issue, the present review has no intention to diminish previous findings and studies; instead, it invites the research community to have in mind the most important differences between both magnetic phases and, hence, their critical determination that is crucial in many applications.

Regarding the polluted agents commonly found in nature and concentrated by human activities, one has to mention the lead divalent, Pb (II), nonionic trivalent arsenic, As (III), and ionic pentavalent arsenic, As(V), which are inorganic species that are high contaminants found in surface and ground water liberated due to natural and human origins [15,16]. In particular, Bundschuh et al. [17] recently published the seven potential sources of arsenic (As) in Latin America that are currently exhibiting high concentrations of As in surface water, groundwater (μg L^−1^), and sediments (mg kg^−1^). The prevalence in water of both As species will depend on the pH, ionic force, and redox potential [18]. In many countries, people drink and ingest water above the permissible levels given by the World Health Organization (WHO) [19], 10 μg L^−1^. Many strategies have been proposed to uptake this toxic metal from water, some of them including membrane separation, ion exchange, microbiological and photochemical oxidation, and adsorption [18,20]. Among the previous cleaning methods, the adsorption method seems to be the most promising due to its various amounts of new magnetic adsorbents that have been recently prepared [15]. These adsorbents can conjugate metal-oxide with commercial mesoporous materials (hybrids) to improve the adsorption capacity and efficiency at various laboratory conditions [18,20]. Moreover, the removal can be enhanced by conjugating the adsorbent with magnetic nanomaterials [21] and, hence, the strong pulling magnetic force of these materials makes them strong candidates to substantially reduce (i) the As pollution problem [15,16] and (ii) the concentration of the adsorbent in remediated effluents. Binary and ternary magnetic nanocomposites [21,22] are emerging nanohybrid materials with a high removal efficiency and can be obtained in high quantities by using the co-precipitation method, i.e., a technique that can be applied in the scaling up procedure and application at the industrial level. To the best of our knowledge, there has not been a review that deals specifically with the differences of Fe_3_O_4_ and γ-Fe_2_O_3_ NPs based on their magnetic surface configurations and their implications when prepared by co-precipitation and also discussing the simultaneous adsorption removal (heavy metal–heavy metal competition and heavy metal–organic removal) of As and Pb ions in contaminated effluents. Therefore, in this review, we bring recent magnetic hybrid nanoadsorbents (prepared essentially by the co-precipitation method) for As and Pb removal, focused on a multiparametric physicochemical analysis and cost evaluation.

## 2. Synthesis Methods of Magnetic NPs

### 2.1. Co-Precipitation Method

**Figure 1:**
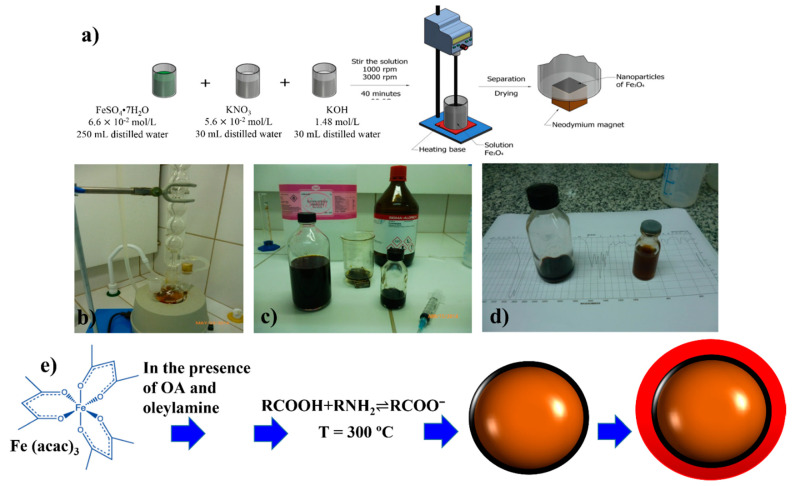
(**a**) Scheme for the co-precipitation method (reproduced with permission from Elsevier [34]). (**b**–**d**) Steps to synthesize Fe_3_O_4_ NPs by thermal decomposition method. (**e**) Biding model of surfactants to Fe_3_O_4_ core. Hydrophobic NPs in organic medium (black layer) and hydrophilic surface modification (red layer). Fe(acac)_3_ indicates the organic precursor of iron (III) acetylacetonate and OA is the oleic acid.

The co-precipitation method consists of a mixture of iron salts, including sulphates, ferric chloride (FeCl_3_), iron (II) chloride (FeCl_2_), or nitrates (FeNO_3_) that precipitate in a high alkaline medium (pH that varies between 10.0 and 12.0), as schematically displayed in Figure 1a. The pioneer in using the co-precipitation method was Massart, who, in 1981, prepared magnetic particles (12 nm in mean size) in an acid and a basic medium without the use of stabilizers [23]. Kinetic factors that influence the nucleation and growth of the iron-oxide crystallites are the most important in the co-precipitation synthesis. The influence of bases in the nanoparticle growth mechanism, such as ammonium hydroxide (NH_4_OH), sodium hydroxide (NaOH), and methylamine (CH_3_NH_2_), were systematically studied by Jolivet in 2004 [24]. Roth et al. [6] also studied the influence of several physicochemical factors in the synthesis of iron-oxide NPs.

The major advantage from the co-precipitation method is the big quantities, in terms of mass, that can be produced, i.e., it is an important factor for technological and environmental purposes, and the major drawback is the size-controlled particle size distribution in a limited range from 3 to 20 nm. However, this issue can be solved by adding either other inorganic or organic agents to easily control the size below 10 nm. According to the literature, this particle size is three times below the magnetic critical diameter established by the formation of magnetic multi-domains for Fe_3_O_4_ and γ-Fe_2_O_3_ [25]. On the other hand, it should be pointed out that other methods (e.g., mechanical milling) can produce NPs with sizes about 13 nm (mean particle size) only after 90 h of milling [26], which may make this method inefficient for scale-up processes.

Some important additional details about the co-precipitation route are:

1. The solution of FeCl_2_⋅4H_2_O has the complex $\mathrm{Fe}{\left[H_{2}O\right]}_{6}^{2+}$, which gives the solution a green-like color.

2. The process of co-precipitation is achieved by adding NaOH to the solution to achieve a high alkaline pH, following the reaction:

(1) $$\mathrm{Fe}{\left[H_{2}O\right]}_{6}^{2+}+\mathrm{Fe}{\left[H_{2}O\right]}_{6}^{3+}+\mathrm{NaOH}\to \mathrm{Fe}{\left(\mathrm{OH}\right)}_{2}+\mathrm{Fe}{\left(\mathrm{OH}\right)}_{3}\to {\mathrm{Fe}}_{3}O_{4}↓$$

This reaction is spontaneous, and the obtained precipitate is $\mathrm{Fe}{\left(\mathrm{OH}\right)}_{2}$ salt, which is poorly soluble in water [27]. Jolivet et al. [28] proved that when the molar ratio ${\mathrm{Fe}}^{2+}:{\mathrm{Fe}}^{3+}$ is 1:2, the chemical reaction is almost immediate at room temperature (RT), being a small fraction of Fe^2+^ sufficient to crystallize all the Fe into an Fe spinel structure. On the other hand, Gokon et al. [29] confirmed that the reaction mechanism with a molar ratio of ${\mathrm{Fe}}^{2+}:{\mathrm{Fe}}^{3+}=$ 1:2 is given by:

(2) $$\mathrm{Fe}{\left(\mathrm{OH}\right)}_{2}+\mathrm{Fe}{\left(\mathrm{OH}\right)}_{3}\to {\left[\left({\mathrm{Fe}}^{3+}\right){\left({\mathrm{Fe}}^{2+}\right)}_{2}\left({\mathrm{OH}}^{-}\right){\left(O^{2-}\right)}_{2}\right]}^{2-}+2H_{2}O\to {\mathrm{Fe}}_{3}O_{4}↓+H_{2}O$$

During the reaction, an unstable intermediate green color solution is obtained. In the case of Fe^2+^: Fe^3+^ = 1:2, the intermediate product is FeO(OH)(s-FeOH^+^), which transforms into Fe_3_O_4_.

As suggested by Equations (1) and (2), the water consumption is an important fact to overcome in the co-precipitation method by underlying its use in the industry, especially in NP washing procedures that often require subsequent steps. It should also be noted that the Fe-oxide NPs, prepared by this method, may frequently present -OH groups at the particle surface that can be avoided using ethanol in the washing procedure.

Regarding the magnetic properties obtained by the co-precipitation method, the NPs can be obtained in three forms: (i) magnetic nanocomposites, (ii) core–shell-like nanostructures, and (iii) magnetic solid matrices. As expected, interesting new magnetic effects, which are still a source of discussion in the scientific literature, appear in each of these configurations due to the predominance of magnetic surface effects.

In particular, novel magnetic properties were observed in ethylenediaminetetraacetic acid (EDTA) functionalized nano-γ-Fe_2_O_3_ prepared by co-precipitation [30]. Three co-precipitation routes were established to synthesize the materials with sizes no bigger than 10 nm, but also no smaller than 4 nm. Then, EDTA acted as a controlled-size reagent that also favors new surface magnetic configuration; consequently, favoring the existence of an exchange bias field that strongly depends on the EDTA shell thickness. In brief, it was experimentally proven that surface (or interface) tailoring by co-precipitation is suitable to observe other magnetic effects [30]. Another important magnetic property found in Fe-oxide NPs when these NPs are embedded in solid matrices (mesoporous and microporous adsorbents) is the superspinglass state not fully comprehended [31].

### 2.2. Thermal Decomposition Method

This method seems to be the most reliable at the time to synthesize pure stoichiometric Fe_3_O_4_ NPs with sizes below 10 nm, a narrow particle size distribution (PSD) and with exotic particle geometries. Some researchers have demonstrated to tune the size and shape of Fe_3_O_4_ NPs by using several solvents with different boiling points [4,32]. The most common precursor is iron (III) acetylacetonate, Fe(acac)_3_, that is thermally decomposed in the presence of 1-octadecene and the surfactants oleic acid and oleylamine (controlling size reagents). The thermal decomposition method frequently requires several steps [33]: (i) temperatures (ca. 300 °C) higher than those used in the co-precipitation process (range of 60–80 °C), (ii) expensive organic precursors and solvents, (iii) long synthesis periods, (iv) careful laboratory conditions, and (v) a subsequent exchange transfer mechanism to obtain high hydrophilic Fe-oxide NPs. However, significant monodispersity can be achieved by the careful handling of the procedures, as described in Figure 1b–e. In addition, the possibility of tuning the morphological appearance of Fe-oxide NPs may also allow to handle and tune the magnetic properties (by controlling the magnetic shape anisotropy). For example, Sun et al. [4] made progress in controlling the exchange bias behavior in core–shell FeO/Fe_3_O_4_ NPs. Their systems (~35 nm) were synthesized by high temperature decomposition of Fe(acac)_3_ in the presence of oleic acid and oleylamine at 300 °C. An exchange bias field of −226 (3) mT was obtained for this configuration.

### 2.3. Bulk Effects in Fe_3_O_4_ and γ-Fe_2_O_3_

An excellent reference to study the bulk and granular properties of magnetite is given by Gorski et al. [35]. A typical bulk stoichiometric magnetite is represented by the formula Fe_3_O_4_, whilst a non-stoichiometric is represented by Fe_3−δ_O_4_, where δ is an indicator of an oxidation state. On the other hand, the γ-Fe_2_O_3_ phase, which can be considered as a consequence of Fe_3−δ_O_4_, is one of the first materials most experimentally investigated at the nanometric scale, because it presents important physical phenomena, such as superparamagnetism and magnetic competing effects [36,37,38,39]. In addition, nano-γ-Fe_2_O_3_ exhibits a ferrimagnetic order in the grain core due to the uncompensated spins of Fe ions that occupy the two sub-lattices of the spinel structure [40], i.e., it represents an uncompensation of ions in the unit cells, generating a high Curie temperature of ca. 950 K and a magnetization of approximately 26.5 µB/unit cell or a net magnetic moment of 2.5 μB/unit (2.5 μB/γ-Fe_2_O_3_, where μ_B_ represents the Bohr magneton) [40].

Indeed, the magnetic and spinel structures consist of two sub-lattices corresponding to Fe located in the tetrahedral sites (A) and in the octahedral sites (B) [1]. Generally, the super-exchange magnetic interactions between the A and B sub-lattices are stronger than those that occur within an individual sub-lattice, such as the Fe spins in each sub-lattice which are ferromagnetically aligned, while also being antiparallelly coupled between the sub-lattices in the bulk-like state [40]. According to the preparation and purity of the macroscopic/mass γ-Fe_2_O_3_ (bulk), the saturation magnetization (*M_S_*) is (74–80 emu g^−1^) and the coercivity (*H_c_*) can be found in the interval between 50 and 800 Oe at RT. If one compares it with other Fe-oxide compounds, the *M_S_* of the γ-Fe_2_O_3_ phase turns out to be less than that of Fe_3_O_4_ (92 emu g^−1^), but greater than that of α-Fe_2_O_3_ (0.1–0.4 emu g^−1^).

## 3. Discussion about Main Differences Based on Physical Techniques

### 3.1. Can the XRD Technique Allow to Differentiate a Nanomagnetite or Nanomaghemite?

**Figure 2:**
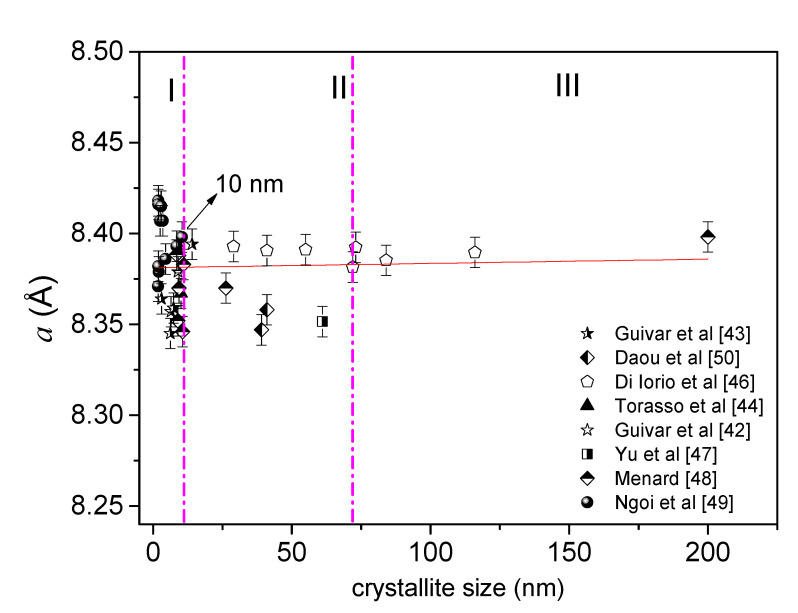
Lattice parameter *a* (Å) vs. crystallite size (nm) for several Fe-oxide NPs prepared by co-precipitation. Figure developed by the authors.

It can be found through a simple search in the literature that the most applied technique to study structural properties and to characterize Fe-oxide NPs is still the conventional XRD method. However, ***is it possible to differentiate with conventional XRD experiments an Fe_3_O_4_ from a γ-Fe_2_O_3_ at a nanoscale regime***? A careful analysis of data published in the literature led us to conclude that the answer, without a doubt, is negative. We can say that both inverse cubic spinel structures are similar, but not identical. The main reason is the presence of vacancies in the bulk γ-Fe_2_O_3_ structure, while the Fe_3_O_4_ phase has both tetrahedral sites fully occupied with Fe(III) spin states and octahedral sites fully completed with Fe(II) and Fe(III) spin states. Thus, the general formula to differentiate them is FTete3+[FOcte1−3δ2+Fe1+2δ3+□δ]O4, where □  is related to the vacancies in the inverse cubic spinel structure [41,42,43,44,45]. Gorski et al. [35] analyzed the unit cell length (a) parameter for different nano-Fe_3_O_4_ as a function of the Fe(II)/Fe(III) ratio. As it can be observed in Figure 2, the lattice parameter of cubic conventional cell fluctuates between *a* = 8.33 and 8.40 Å, where a total oxidized γ-Fe_2_O_3_ will depict a value of approximately *a* = 8.34 Å. It is important to highlight that the particle size reported in Gorski et al.’s work [35] was of 20 nm, and the current sizes required for many nanotechnological applications must be below this value. More importantly, Fe-oxide NPs with smaller sizes will have uncompensated spins at the particle surface; consequently, a surface disorder layer occurs (often not detectable by conventional XRD experiments), leading to interesting magnetic properties due to finite-size effects. Therefore, Figure 2 summarizes and shows an intrinsic correlation between the lattice cubic parameter (a) values and crystalline grain sizes reported in the literature found in the spinel-like structures (the crystallite size values were obtained by the refinement of XRD data [42,43,44,46,47,48,49,50]). In a first approximation, it can be noticed that the values of lattice parameters for both Fe-oxide phases (Fe_3_O_4_ and γ-Fe_2_O_3_) are similar at the nanoscale, making the identification difficult. However, three different regions can be identified for all the Fe-oxide NPs: (i) the first (I) region, with sizes below 10 nm, shows a variation of the parameter *a* (Å) values in the range of 8.34 to 8.43 Å, confirming the difficulty in differentiating between nano-Fe_3_O_4_ and nano-γ-Fe_2_O_3_; (ii) values of *a* parameter between 8.35 and 8.39 Å were obtained for grain sizes from 10 to 70 nm (region II); for the third (III) region, a constant value of 8.39 Å was obtained, suggesting the predominance of the Fe_3_O_4_ atomic arrangement in high crystallite sizes. Moreover, a positive linear trend (red full line obtained from a fit) can be considered around for data in regions II and III, as suggested by Figure 2. Additionally, Schwaminger et al. [51] tried to differentiate between nano-Fe_3_O_4_ and nano-γ-Fe_2_O_3_ based on the analysis of the (440) Miller plane of an Fe-oxide sample prepared by co-precipitation. However, two problems can be pointed out: (i) no control in oxidation was conducted during the Fe-oxide NP’s synthesis and (ii) the Scherrer method was used to estimate nanocrystallite diameters (using only one diffraction peak). The latter permits to mention that this integral breadth method is known to overestimate the real crystallite size, and the Rietveld method must be used for a better estimation. No other technique (e.g., Mössbauer spectroscopy) was discussed to prove the presence of pure nano-Fe_3_O_4_. On the other hand, González-Alonso et al. [52] recently studied the presence of Fe_3_O_4_ in an ensemble of 28 nm Fe-oxide NPs by means of neutron diffraction. The authors discussed the presence of nano-Fe_3_O_4_ for samples with mean sizes higher than 10 nm, where significant clues (agglomeration) of nano-Fe_3_O_4_ are experimentally observed. From the structural point of view, it can be concluded that conventional XRD experiments can hardly bring any trustable information of the Fe-oxide phases (e.g., a Fe_3_O_4_ from a γ-Fe_2_O_3_) in a nanoscale regime, at least, for grain sizes smaller than 10 nm (region I of Figure 2).

### 3.2. Mössbauer Technique as the Main Tool of Differentiation

**Figure 3:**
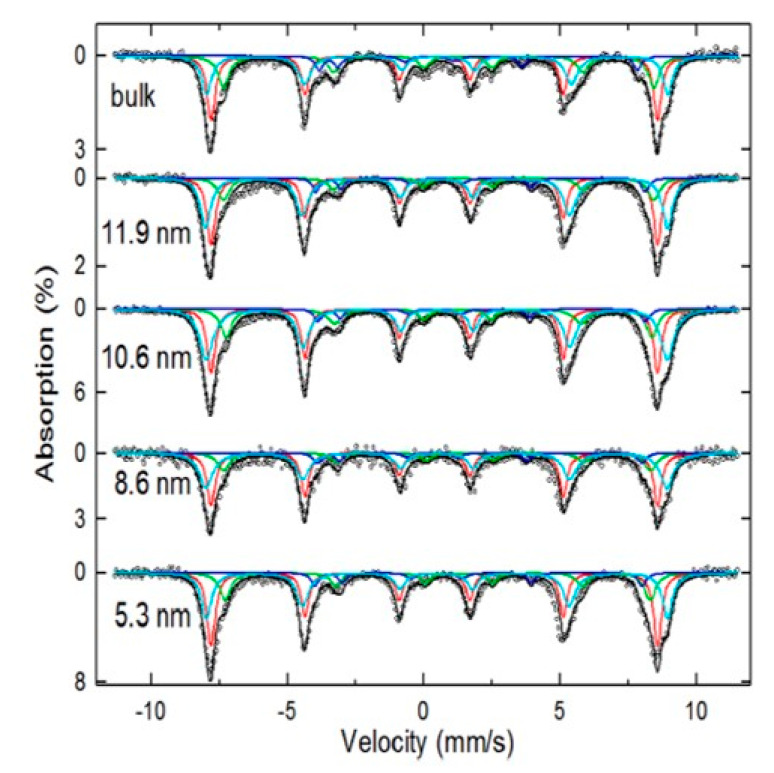
Mössbauer spectra of Fe_3_O_4_ at 6 K of bulk and 11.9 nm, 10.6 nm, 8.6 nm, and 5.3 nm particles. Fitted spectra are (red) tetrahedral Fe(III), (light blue) octahedral Fe(III), (green and dark blue) octahedral Fe(II). (Reproduced with permission of Elsevier [7].)

Often, a bulk γ-Fe_2_O_3_ phase has been reported to have an asymmetric magnetic spectrum with narrow adsorption lines characteristic of two static magnetic components, represented, respectively, by site A (37.5%) and site B (62.5%) of the spinel-like structure [30]. For instance, Tuček et al. [13] reported the difference between bulk γ-Fe_2_O_3_ and nano-γ-Fe_2_O_3_ from the perspective of Mössbauer spectroscopy. The authors took the case of ideal superparamagnetic behavior as described by the ideal blocking temperature ($T_{B}$) relation:

(3) $$T_{B}=\frac{KV}{k_{B}\ln\left(\frac{\tau_{m}}{\tau_{0}}\right)}$$

where *K* is the effective anisotropy constant, $k_{B}$ is the Boltzmann’s constant, *V* is the particle volume, $\tau_{m}$ is the characteristic time of the technique, and $\tau_{0}$ is the relaxation time constant. Equation (3) uses the case of monodisperse nano-ensembles with a narrow particle size distribution (PSD), anisotropic energy distributions and Fe-oxide NPs non-interacting magnetically. In the case of Fe-oxide NPs with a broad PSD, the Mössbauer analysis is more complex if one considers that the spectra will result in a combination of several components due to the spin relaxation and spin disorder surface effects. However, in general, the shape of Mössbauer spectra of magnetic NPs at 300 K can be used to classify the PSD in two different categories: (i) if the samples have a broad PSD, the Mössbauer spectra will have a mixture of relaxed superparamagnetic and magnetic components with broad adsorption lines. The Fe-oxide NPs responsible for this complex spectrum will have mean sizes of 10 nm or higher and the average blocking temperature (*T_B_*) will usually be above 300 K [12,41,53]; (ii) if the Mössbauer spectrum at room temperature (RT) shows only a strong relaxed component (only a doublet or singlet corresponding to a superparamagnetic regime), a relatively narrow PSD can be assumed and, in addition, the interparticle magnetic interactions are either missing or weak. In the latter case, the samples must have Fe-oxide NPs with grain sizes smaller than ~8 nm and the values of *T_B_*, in general, will be below 300 K (the *T_B_* value will depend on the effective magnetic anisotropy constant and also the average volume of the particles) [50,54]. Furthermore, it should be mentioned that a nano-Fe_3_O_4_ also exhibits extra absorption lines with an inverted spectrum as compared to nano-γ-Fe_2_O_3_ [7]. These extra absorption lines at a low velocity are less intense and characteristic of a nano-Fe_3_O_4_ structure [7]; see Figure 3 (green sextet).

Taking results from the literature, it can be indirectly inferred that Mössbauer spectroscopy has often been implemented in Fe-oxide NPs with sizes bigger than 10 nm [8,11]. As an example, da Costa et al. [12] studied the magnetic properties of nano-Fe_3_O_4_ and nano-γ-Fe_2_O_3_ prepared by wet chemical methods and, using integral low-energy electron Mössbauer spectroscopy (ILEEMS), they were able to differentiate between both nano-Fe-oxides with grain sizes of 15 nm (Fe-oxide NPs with sizes smaller than this value were not reported). Nedkov et al. [3] also studied the magnetic properties of nano-Fe_3_O_4_ synthesized by co-precipitation. Two samples with mean sizes of 10 (2) nm and 3 (2) nm were prepared and studied by ILEEMS. It was concluded that both nano-Fe_3_O_4_ samples had a contribution of γ-Fe_2_O_3_ on the sample surfaces. For samples with smaller particle sizes, a contribution of 30–40 vol% of the total Fe_3_O_4_ core was observed, but the authors did not show a 4.2 K Mössbauer spectrum to corroborate the presence of Fe_3_O_4_. A similar spectrum at RT was reported in [43] and it was attributed to a pure γ-Fe_2_O_3_ based on the resolved Mössbauer spectrum at 12 K. Therefore, it is not possible to affirm from an RT Mössbauer spectrum that the sample is nano-Fe_3_O_4_ or nano-γ-Fe_2_O_3_, mainly because of the magnetic relaxation contribution, and also due to the fast oxidation of Fe(II) ions to Fe(III) at ambient conditions. Even in functionalized samples prepared by co-precipitation, a fast oxidation may also occur. Some examples are given in [3,4,8,9,30,43,55,56,57] for inorganic and organic coatings.

**Figure 4:**
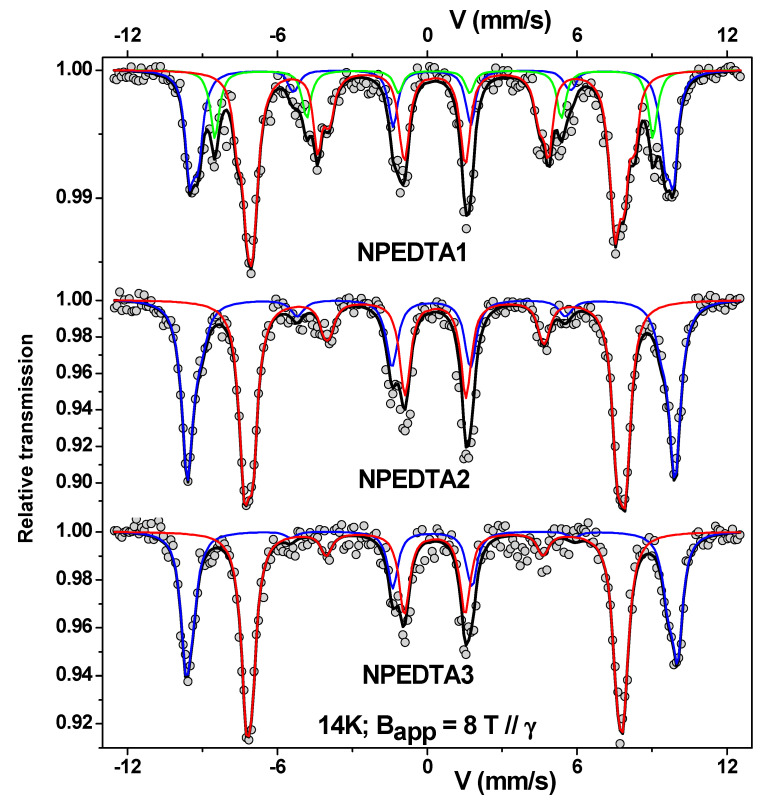
In-field Mössbauer spectra taken at 14 K in an applied field of 8 T for the ethylenediaminetetraacetic acid (EDTA) functionalized γ-Fe_2_O_3_ NPs (NPEDTA). Mössbauer spectra of the NPEDTA2 and NPEDTA3 were reproduced with permission of Elsevier [30]. 1, 2, and 3 indicates different routes of synthesis that can be checked in [30]. The lines red and blue represent the crystalline sites of γ-Fe2O3 NPs while the green line for NPEDTA1 sample is related to the surface magnetic contribution.

As discussed above, either the XRD or zero-field ^57^Fe Mossbauer spectroscopy will hardly differentiate the nano-Fe_3_O_4_ or nano-γ-Fe_2_O_3_ when the sizes are below 10 nm due to the Fe spin relaxation and spin surface disorder effects. To overpass this point, low temperatures, and in-field Mössbauer measurements are necessary. In other words, by applying an external magnetic field, the corresponding magnetic sites align to the external field direction, resolving the magnetic relaxation processes and the Fe atomic coordination and spin configurations. Thus, with this information, and assuming the different isomer shift (δ) values of these two Fe-oxide phases, one can more easily separate their contributions in samples for in-field Mössbauer experiments since it will allow to know the proportions of relative absorption areas for each magnetic site, as shown in Figure 4 and in Table 1 for samples of the pure nano-γ-Fe_2_O_3_-like phase that were EDTA functionalized. In addition, it was possible to demonstrate the fractions and the canting angles of Fe spins on the particle surfaces by performing in-field Mössbauer data, correlating, for example, with the thickness of the EDTA layer on the nano-γ-Fe_2_O_3_ NPs. Table 2 summarizes the main values for the hyperfine parameters of Fe_3_O_4_ and γ-Fe_2_O_3_ in both bulk and nanoscales. Therefore, to solve the problem associated with the nano-Fe_3_O_4_ and nano-γ-Fe_2_O_3_ phases, Mössbauer experiments under high fields and low temperatures are required.

### 3.3. High Resolution XPS and Synchrotron Radiation Techniques

XPS has been employed for surface (few nanometers) characterization of functionalized Fe-oxide NPs. For example, Wilson et al. [55] investigated the stabilization of oleylamine/oleic acid-capped Fe_3_O_4_ NPs. The characteristic low energy peak at 710.2 eV was employed to identify Fe(II), while the peak located at 710.8 eV related to Fe^3+^ octahedral species, was observed. Despite the identification of both valence states, the 2p_3/2_/2p_1/2_ ratio was found equal to 1.7, a value that is close to that found in the ideal stochiometric Fe_3_O_4_ (ratio of 2). To account this difference, the authors suggested an additional contribution of Fe^3+^ coming exclusively from the surface due to the presence of γ-Fe_2_O_3_ NPs. On the other hand, Lavorato et al. [8], using XPS measurements, studied the 12.1 nm ˈFe_3_O_4_ˈ NPs coated with Zn_0.6_Fe_2.4_O_4_ for as-prepared and 6-month aged samples. They identified three Fe 2p_3/2_ energy bands at 710.1, 711.5, and 713.8 eV related, respectively, to Fe(II) and Fe(III) in the octahedral and Fe^3+^ in the tetrahedral sites. In addition, the ratio Fe(II)/Fe(III) was found to be equal to 0.39 for the as-prepared and 0.31 for the aged samples, suggesting a gradual oxidation of the particle surface. A similar oxidation behavior was observed by Bhattacharjee et al. [9], where a satellite peak at 718.08 eV was assigned to the surface peak of the γ-Fe_2_O_3_ phase and related to partial oxidation of the Fe-oxide NP surface. On the other hand, the functionalization of γ-Fe_2_O_3_ NPs with nanohydroxyapatite, as obtained by the co-precipitation technique, was investigated by Guivar et al. [41] and the XPS results showed the Fe_2_p_3/2_ at 710.7 eV related to Fe(III) in a total oxidized γ-Fe_2_O_3_. However, as can be noticed from Guivar et al. [41]’s paper, the mathematical fit could also be conducted with three peaks assuming the presence of Fe_3_O_4_.

In the resume, this type of analysis obtained from broad lines, in principle, seems to have a strong mathematic character; consequently, the fit model should be carefully conducted and supported by other high-resolution techniques. Based on the above discussions, there is an important issue that still deserves to be stressed: ***when is the exact time that a transformation from nano***-Fe_3_O_4_ ***to nano-***γ-Fe_2_O_3_ ***occurs?*** According to results reported in the literature, the thermal decomposition method allows a better control of the surface oxidation process as compared to the co-precipitation method. Considering that the nanomaterials have a large active surface, the oxidation process will occur instantly, forming a core–shell-like nanosystem; therefore, it is hard to believe that fully stoichiometric nano-Fe_3_O_4_ NPs can be prepared, as has been claimed in the literature several times [3,8,9,55,56,57]. One possibility to solve this point is to perform experiments in high resolution facilities, such as a synchrotron light to probe spin structures of different samples. As an example, high energy resolution techniques, such as X-ray absorption spectroscopy (XAS) and X-ray magnetic circular dichroism (XMCD) have also been applied to elucidate the magnetic properties of Fe-oxide core–shell nanosystems. Jiménez-Villacorta et al. [56] reported that Fe-oxide NPs composed of 70% Fe_3_O_4_ and 30% γ-Fe_2_O_3_ exhibited a magnetic signal at 1 kOe and 25 K, suggesting the formation of a core–shell system. Moreover, Bonanni et al. [57] studied the effect of a low-field (160 Oe) XMCD measurement on the magnetic properties of 13 nm and 7.0 nm hollow γ-Fe_2_O_3_ NPs and 5.0 nm bare NPs. The XAS measurement of the 7.0 nm γ-Fe_2_O_3_ displays the main peak of the FeL_3_ edge (site A) and the double peak at the FeL_2_ edge (site B), i.e., the XCMD spectrum revealed information of site A (positive peak) and site B (two negative peaks) [57]. Additionally, the bare and 13 nm γ-Fe_2_O_3_ NPs showed the same prevalence of Fe(III) spins, but a reduction in the magnetization was observed for the 7.0 hollow NPs and related to the formation of a frustrated magnetic state at the particle surface. Anyhow, XPS and XMCD can be applied to bring information about the Fe valence state and also some information about the magnetism of the Fe-oxide NPs, but these techniques must also be applied simultaneously with the in-field Mossbauer one. Therefore, to understand the adsorption process, we have to characterize the sample deeply and the atomic and spin configurations must be raised up. From now on, we can start to discuss individually the adsorption process of As and Pb using the Fe-oxide NPs based on published results. For that, we analyze different experimental conditions (pH, dose, temperature, etc.).

## 4. In-Detail Discussion of the Adsorbent Properties

### 4.1. As Adsorption Experiments

#### 4.1.1. Individual As Adsorptive Properties

Considering water magnetic remediation processes, it is of great interest to know the correct surface configuration of the Fe-oxide NPs from the atomic arrangement and magnetic point of view as presented in Table S1 (see Supplementary Material). Indeed, many magnetic Fe-oxide NP composites have been tested as As adsorbents in recent years [58,59,60,61,62,63,64,65,66,67,68,69,70,71,72,73,74,75,76,77,78,79,80,81,82,83,84,85,86,87,88]. Their adsorbent properties and experimental conditions are exposed and a comparison of their removal efficiency and an analysis of the experimental condition effects (pH, temperature, adsorbent dose, initial concentration, particle size and shape, surface area, and saturation magnetization, equilibrium time, kinetic and isothermal adsorption parameters) [58,59,60,61,62,63,64,65,66,67,68,69,70,71,72,73,74,75,76,77,78,79,80,81,82,83,84,85,86,87,88] on the As removal are of great importance and are summarized in detail in Table S1. The main results suggest that the temperature has been a decisive factor in the adsorption removal efficiency and more specifically, the adsorption capacity rose with the temperature in several cases [69,72]. However, there also are special cases where the adsorption capacity first increases with the temperature, but it decreases, subsequently [58], indicating that the effect of temperature influences on As removal for all adsorbents (shown in Table S1) has still not been solved. In brief, various adsorbents have shown different temperature dependences in their performance in agreement with results published by Siddiqui et al. [89]. However, room temperature has been chosen by many authors due to its commonness in nature and because this temperature usually exhibits a good removal performance, reducing the cost of the entire process. The effects of pH on As(V) adsorption have been mainly attributed to the protonation and deprotonation of the adsorbent’s surface below and above the p.z.c., respectively [42,60,62,63,64,66,70]. This effect is due to the negative charge of As(V), which engages in an electrostatic interaction with the surface radicals of the adsorbents. Another relevant factor is the radical’s variability with pH of the adsorbates and adsorbents, although it contributes more to As(III) removal than to As(V) removal due to the As(III) charge neutrality [47,59,66]. As reported by Das et al. [66], the number of surface hydroxyl groups increases with the pH augments and, therefore, contributes towards the enhancement of the As(III) removal at a higher pH. In other words, the As species change when the pH increases and, thus, the number of hydroxyl groups increases, raising the As(III) adsorption effectiveness [59].

In a first phenomenological description of As adsorption processes, the Langmuir adsorption model is the most used one [42,60,64,65,70,75], while the Freundlich adsorption model can be considered as the second most applied [63,68,71] for both As(III) and As(V). For these reasons, it can be indirectly inferred that the monolayer adsorption mechanism is used more often than the multilayer adsorption. However, it must be noticed that Nisticò et al. [73] and Yu et al. [47] obtained better Langmuir model R^2^ values for As(III) adsorption, whereas a better fitting with the Freundlich model for As(V) adsorption was reported by Nikić et al. [74] and Zeng et al. [76]. The above results suggest that multilayer adsorption is dominant in the case of As(V) ions as compared to As(III). In addition, the Sips isotherm adsorption model, which consists of Langmuir and Freundlich models, was successfully applied to fit the adsorption isotherms of MBC [59], Fe_2_O_3_-ZrO_2_/BC [68], and nano-γ-Fe_2_O_3_-TiO_2_-GO nanohybrids [42]. It should be stressed that the Sips model is often used when no maximum adsorption saturation is reached, and the nano-adsorbent can continue capturing the heavy metal at higher concentrations.

To give an example of the As adsorption process by Fe-oxide NPs, we bring the work of Siddiqui et al. [58] which found that at 35° C and 45 °C the Freundlich model fitted the adsorption isotherms of the nano-γ-Fe_2_O_3_@starch better than the Langmuir model, while for the non-functionalized nano-γ-Fe_2_O_3_ the opposite happened. These findings could indicate that starch functionalization and temperature raising may enhance multilayer adsorption. Another important parameter in the As removal process is its concentration in the synthetic effluent. Navarathna et al. [59] measured the As(III) removal percentage at a constant adsorbent dose (50 mg) and concentrations of 5, 10, and 20 mg L^−1^ (25 °C, pH = 7.0). Navarathna et al. [59] found that: (i) the As percentage reached its highest value at 20 mg g^−1^ and (ii) it increased from 5 to 10 mg g^−1^ due to the multilayer adsorption character that occurs on the nano-Fe_3_O_4_ surface. Raval et al. [63] also found that increasing the As(V) concentration from 10 μg L^−1^ to 150 μg L^−1^ lowered the adsorbed As percentage due to the saturation of available adsorption sites in the bilayer–OA@FeO NPs adsorbent. Moreover, they also observed that the adsorption capacity increased due to the high driving force to transfer the mass of elevated concentrations of As(V) in the solution, as observed in the adsorption isotherms. Briefly, considering the As adsorption process, the adsorbents that exhibited the best performances and their maximum adsorption capacities in mg g^−1^ according to the Langmuir adsorption model, presented for both As species, and compared to other systems [58,59,60,61,62,63,65,66,68,69,70,71,72,73,74,75,76,77,78,79,80,81,82,83,84,85,86,87,88,89,90,91,92,93,94,95,96,97,98,99], were FeO_X_-GO-80 with 147 mg g^−1^ (arsenite) and 113 mg g^−1^ (arsenate) [64], γ-Fe_2_O_3_@CTF-1 with 198 mg g^−1^(arsenite) and 102.3 mg g^−1^ (arsenate) [67], and γ-Fe_2_O_3_-TiO_2_-GO with 110.4 mg g^−1^ (arsenite) and 127.2 mg g^−1^ (arsenate) [42].

#### 4.1.2. Effect of pH in the Independent Removal of As(III) and As(V)

pH is definitely another important factor in the adsorption As removal experiments. In the solution with a pH ranging from 3.0 to 9.0, the charge neutral H_3_AsO_3_ is the dominant species of As(III), whereas for As(V), the negatively charged H_2_AsO_4_^−^ and HAsO_4_^2−^ are the most prevalent forms [92,100]. The shifting of the pH_p.z.c._ value indicated specifically the As(V) adsorption rather than electrostatic interactions. Therefore, the formation of complexed/precipitated As species at the surface of the nano-Fe_3_O_4_ adsorbent is one of the main adsorption mechanisms [93,101,102]. The *p*Ka values of As acid are *p*Ka_1_ ≈ 2.3, *p*Ka_2_ ≈ 7.0, and *p*Ka_3_ ≈ 11.5, indicating that the molecular form mainly exists in a solution at a pH < 2.0, while the anionic species (H_2_AsO_4_^−^ or HAsO_4_^2−^) exists at a pH in the interval 2–10 [14]. At pH < pH_p.z.c._, the protonation of surface functional generates a positive charge, which contributes to the favorable bonding of negatively charged arsenate ions. Both surface states and As speciation play significant contributions to electrostatic interactions (attraction/repulsion) between surface/ions, causing the intensity of the As to flux towards the specific adsorption sites. The enhancement of electrostatic attractions is, thus, highly feasible for As(V) species, while it is of minor importance for the neutral form of arsenous acid [103]. The pH also affects the surface charge of the adsorbent NPs. The surface of the adsorbent is positively charged when the equilibrium pH is below pH_p.z.c._ and, consequently, negatively charged when the equilibrium pH is above pH_p.z.c._ [96]. For instance, Narouei et al. [90] demonstrated that the adsorption of As with Fe_3_O_4_ NPs decreased by 30% when the experiments were carried out at a pH ca. 9; thus, the authors decided to perform most of the experiments in a pH of 7.5 that is a representative pH of most aquatic environments. In their research, humic acid (HA) has a greater structural complexity that makes it soluble at a high pH and insoluble in acidic conditions. The oxidation signals of As(III)/As(V) indicate that under these conditions (pH 6–8 and 0–10 mg L^−1^ HA), the As adsorbed in Fe-oxide NPs is predominantly present as As(III) and that any possible chemical oxidation by Fe_3_O_4_ would also imply the reduction from the newly formed As(V) back to As(III). Compared to the two mentioned cases, the reduction in As(V) to As(III) or probably the limitation of the initial oxidation of As(III) is more effective when HA is present. HA adsorption was found to increase the stability of NPs dispersions over a wide pH range and prevent salt-induced aggregation at a neutral pH [104]. Results from Paul et al. [91] agreed with the previous study when they investigated the influence of the pH dependence of HA on surface charges and found that in the absence of HA, which covers the surface charge of graphene oxide-Fe (GO-Fe), the p.z.c was −34.8 mV at pH 7.0. After coating with HA, the composite showed almost double the removal efficiency of As(III) and As(V) at a neutral pH. To comprehend the positive role of HA towards As adsorption, Paul et al. [91] performed zeta potential measurements on all samples, which led them to envision the role of the pH and surface charge of HA dynamics with GO in the nano-Fe_3_O_4_ composite for As removal. Rashid et al. [92] also reported the adsorption of toxic inorganic As species from aqueous onto HA grafted Fe_3_O_4_ NPs (HA-MNP) in the pH range of the solution from 3.0 to 9.0. At a higher pH, As(III) ions are more easily converted to As(V), which may be related to *p*Ka, changes in speciation, and the susceptibility to oxidation. Despite electrostatic repulsions, chemical reactions between the functional groups of HA and As(V) appear to be dominant in the adsorption process. Yoon et al. [96], who investigated the adsorption of As(III) and As(V) on Fe_3_O_4_/non-oxidative graphene (M-nOG) composites, reported that at a pH ranging from 4.0 to 10.0, the As speciation is significantly modified. It should also be stated that Fe-oxide NPs, such as Fe_3_O_4_, typically have pH_p.z.c._ values in the range of 7–9 [105]; therefore, relevant for the As removal process. In Yoon et al.’s study [96], the pH_p.z.c_. value of M-nOG was determined to be pH 7.1. However, when the pH increased, the adsorption of As(V) on M-nGO decreased, because of the negatively charged surface sites on the adsorbent and increased competition between hydroxide ions (OH^−^) on As(V). With this interesting observation, Yoon et al. [96] indicated that the electrostatic interactions between the positively charged surface of M-nOG and anionic As(V) species are one of the major factors for As(V) removal.

#### 4.1.3. As Adsorption Mechanism and Adsorption Isotherm Models

In the research carried out by Rashid et al. [92], the results of removal showed that As(V) is adsorbed by HA-MNP faster and to a greater extent than As(III). A dose of HA-MNP of 0.2 g L^−1^ and an initial concentration of 200 μg L^−1^ of each species of As (As(III) and As(V)) were used. From these experiments, it was possible to reduce the level of As(III) below the drinking water maximum contaminant level of 10 μg L^−1^ in 180 min, while only 60 min are required to decrease the As(V) concentration below the maximum contaminant level. Within one min of exposure to HA-MNP, the initial concentrations of As(III) and As(V) reduced by more than 50%. So, this study indicates that the adsorption of As(III) and As(V) occurs in three different stages within the functionality of the HA coating via surface association, intraparticle diffusion, and complexation reactions or ligand exchange [92]. The initial As concentration was varied from 0.1 to 10 mg L^−1^ at a constant loading of HA-MNP (0.2 g L^−1^) and the adsorption process was found more consistent with the Freundlich model, indicating that the adsorption occurred in a multilayer formation and/or heterogeneous adsorption surface sites of the HA-MNP. From the Freundlich model, the value of 1/n less than one was an indicative of chemisorption of As(III) and As(V) on HA-MNP. Taleb et al. [93] used adsorbents containing cellulose support (MC) versus nanocellulose (NC) for the preparation of nano-Fe_3_O_4_ (referred as MG in [93]) and studied the As removal. The results indicated that the most acceptable fitting model should be conducted using the Langmuir and Freundlich isotherm models. Somewhat, the capacity of the As(III) removal of 68.2 and 17.8 mg g^−1^ was obtained with NCMA/L-MG and MC-O/L-MG, respectively. On the other hand, Lung et al. [95] found that the isotherms of the Langmuir and Freundlich models were the most suitable to describe the adsorption process of Pb(II), Cd(II), and As(III) on green Fe_3_O_4_ NPs. The adsorption energy (bT) was positive for all metal ions in the liquid matrix, indicating that the adsorption is an exothermic process [95]. In the study conducted by Yoon et al. [96], who studied the As(III) and As(V) removal using M-nOG composites, they found that the Sips isotherm model fit better the experimental data in comparison to the Langmuir and Freundlich models. This means that the As adsorption mechanism of the M-nOG hybrid is adequate for the combined Langmuir–Freundlich isotherm. In another research [97], the As(V) removal presented in the drinking water level was removed by using sand coated with nano-Fe_3_O_4_. The batch experiments were carried out in the presence of coexisting cations (Zn(II), Cd(II), Pb(II), Ni(II), Mg(II), Cr(III), and Fe(III)) to understand its impact on the removal efficiency of As(V). The As adsorption data were fitted using the Langmuir and Freundlich models, obtaining an R^2^ value of 0.99 (1) for both models. Similar to the previous works [96,97], the adsorption of As(V) with nano-γ-Fe_2_O_3_ and graphene oxide (GO) embedded in the polyacrylonitrile (PAN) polymer nanofibers matrix was also studied [98] on the basis of the Langmuir and Freundlich isotherm models. According to the Freundlich isotherm model, the maximum adsorption capacity was 36.1 mg g^−1^. An important point to be highlighted related to As removal is the fact that the adsorption efficiency was not affected by the presence of Cl^−^, NO_3_^−^, and SO_4_^2−^ ions, but with PO_4_^3−^ ions that reduced significantly the efficiency of adsorption [98].

#### 4.1.4. Effect of Organic Pollutants on the As Simultaneous Uptake

The organic matter present in natural water ecosystems, as humic substances (HS), are between 0.1 and 20 mg L^−1^ [90]. They are produced from the decomposition of soil humus and various aquatic plants through various biological and chemical processes. HA is the main constituent and shows the most hydrophobic and high molecular weight fraction. It exhibits high sorption and complexation characteristics compared to other HS fractions [92] and can severely affect the As removal process when Fe-oxide NPs are applied [91,92]. Therefore, when As and HA are present in contaminated water, competitive adsorptions on Fe-oxide NPs can occur, reducing the adsorption of As. For 5 mg L^−1^ HA, Narouei et al. [90] reported a 97% removal efficiency of a 10 µM solution of As, as can be seen in Table S2. However, when HA was prepared at 50 mg L^−1^, the removal efficiency of 10 µM As(III) decreased to 90.1%, whereby As(III) has a lower accessibility to the surface of Fe_3_O_4_ NPs. Additionally, as expected, when the As concentration was 100 M, the As removal efficiency decreased for both HA levels. Paul et al. [91] indicated that the HA coating was influenced by graphene in the nano-Fe_3_O_4_ composite, which turned out to be an enhancing effect in the removal of As from contaminated effluents. After coating with HA, the nano-Fe_3_O_4_ composite showed almost double the removal efficiency of As(III) and As(III) at a neutral pH.

#### 4.1.5. Effect of the Coexisting Anions Cl^−^, NO_3_^−^, and SO_4_^2−^ on the As Adsorption

In natural water sources, such as groundwater, several anions can coexist in As, and due to its competitive binding activity, it significantly decreases the percentage of As removal by applying Fe-oxide NPs [96,98]. Tripathy et al. [98] studied the effect of chloride (Cl^−^), nitrate (NO_3_^−^), sulphate (SO_4_^2−^) on the adsorption of As(V) by PAN/GO/-Fe_2_O_3_ nanofibers, showing that these anions have no appreciable effect on As(V) adsorption [98], a result that agrees with the findings of Rashid et al. [92], Yoon et al. [96] and Taleb et al. [93]. On the other hand, there was no inhibition of As(III) adsorption [92,96] even when the adsorption of metal ions (As(III), Pb(II), and Cd(II)) from natural waters was performed. Therefore, As(III) has been retained very well on Fe_3_O_4_ NPs, while the other two metal ions, Pb(II) and Cd(II), were kept in a very small percentage due to the presence of competing metal ions that influence the sorption process [95]. The Cl^−^ and NO^3−^ ions are only adsorbed as diffuse ions on the outer surfaces of the adsorbent [96], even when the As removal is increased due to the binding of negatively charged As species. Therefore, the concentration of negative charge increases in an electric double layer, and the enhancement of the ionic strength of the solution and the higher the concentration of counter cations could compensate the negative charges on the Fe-oxide hybrid surfaces. The observed facts, associated with the increase in ionic strength, lead to a higher adsorption of arsenate, indicating that the main adsorption mechanism is the formation of inner-sphere complexes [93]. On the other hand, sulfate, as divalent ions, forms external sphere complexes with strong electrostatic interactions, resulting in a higher ionic competition during the adsorption process. Finally, the effect of SO_4_^2−^ on As adsorption was relatively high for both As(V) [93], as well as for As(III) and As(V) [96].

#### 4.1.6. Influence of PO_4_^3−^ on the As Adsorption

As-contaminated water containing phosphate anions as co-anions significantly decreased As removal [96,97,98,99]. The major adverse effect shown was the role of the phosphate competitive adsorption of As(V) compared to the coexisting Cl^−^, NO_3_^−^, and SO_4_^2−^ anions [93]. Rashid et al. [92] and Yoon et al. [96] demonstrated that the phosphate competition for HA-MNP and M-nOG active sites, respectively, decreases the adsorption for both As(III) and As(V), although As(III) was slightly more affected. These phenomena may be caused by the fact that phosphorus (P) and As have structural and chemical similarities, as they are both present in the same group 15 (5A) of the periodic table [93,98]. Therefore, they can form inner-sphere complexes through a ligand substitution reaction on the Fe-oxide hybrid surface [96] and, thus, exhibit adsorption and chelation properties similar to As and compete for adsorption on adsorbent sites [92]. Nevertheless, in rivers and groundwaters, the mass ratio between phosphate and As is usually very low, so there would be no major problems [92], as confirmed by an investigation performed by Lung et al. [95] who carried out the removal of As(III), Pb(II), and Cd(II) in a river sample using nano-Fe_3_O_4_ in the presence of anions and cations with a low concentration of PO_4_^2–^. The authors found that the removal of As(III) was optimal, whilst the removal of Cd(II) and Pb(II) was in a very small percentage. This observation may indirectly favor saying that the presence of metal ions competes in the sorption process at all.

#### 4.1.7. Effect of the Coexisting of Metal Ions on the As Removal

As mentioned above, As and metal ions often coexist in an ambient environment of the water system [92]. At pH = 2.3–6.9, the dominant As(V) species is H_2_AsO_4_^−^ [97,105]. Darezereshki et al. [94] studied the influence of Cu(II), Zn(II), and Mn(II) ions on arsenate adsorption for pH < 6, with the highest adsorption rate of As(V) on copper (Cu), zinc (Zn), and manganese (Mn); this was caused because As has a higher electronegativity value compared to Cu, Zn, and Mn, being 2.2, 1.8, and 1.6, respectively. Additionally, the H_2_AsO_4_^−^ species has a low charge density and low hydration capacity, so it can be easily adsorbed on nano-Fe_3_O_4_ due to its high mobility. On the other hand, Cu divalent ions have a better interaction with the negative charge of the adsorbent surface due to its hydrated ionic radius, which is smaller than zinc and manganese ions, being 4.19 Å, 4.30 Å, and 4.43 Å for Cu, Zn, and Mn, respectively. Thus, the order of adsorption of these metals can be assumed as: As(V) > Cu(II) > Zn(II) > Mn(II), even if the presence of cations in the solution can reduce the negative charge of the adsorbent surface and, therefore, facilitate the adsorption of the anionic arsenate. This effect is because the negative surface charge of the adsorbent attracts positive complexes such as copper (CuH_2_AsO_4_^+^), manganese (MnH_2_AsO_4_^+^), and zinc (Zn H_2_AsO_4_^+^) ionic compounds that can electrostatically adsorb on the negative surface of the adsorbent. Kango et al. [97] investigated the effect of individual cations (Zn(II), Cd(II), Pb(II), Ni(II), Mg(II), Cr(III), and Fe(III)) on the adsorption of As(V) with nano-Fe_3_O_4_-coated sand. For the presence of cations with a concentration of 100 mg L^−1^ coexisting in As(V), it showed a little interference with As(V) removal being between 94% and 99%. In particular, the effect of cations on As(V) adsorption efficiency was found to be in the order of Cr(III) > Mg(II) > Ni(II) > Pb(II) > Cd(II) > Zn(II) > Fe(III). The removal of As(III) was also not affected by the presence of inorganic ions in the adsorption with nano-Fe_3_O_4_, even in the presence of other heavy metal ions, as Pb(II) and Cd(II), which were retained in a very small percentage [6].

Darezereshki et al. [94] demonstrated the substitution effects of metal ions (Al(III) and Fe(III)) on arsenate adsorption at pH = 2.0, by the formation of FeH_2_AsO_4_^2+^ and AlH_2_AsO_4_^2+^ complexes with free H_2_AsO_4_^−^ species. They showed that the As(V) removal capacity with nano-Fe_3_O_4_ decreased as the presence of Al and Fe ionic species increased with respect to arsenate. In addition, the presence of Fe(III) ions decreased arsenate adsorption more compared to the Al(III) ions. This observation was explained assuming the higher reactivity of Fe(III) and arsenate species (H_2_AsO_4_^−^), and the higher stability of FeH_2_AsO_4_^2+^. However, the low concentration of Fe(III) ions in the solution did not seem to greatly affect As adsorption [94], which was confirmed by Rashid et al. [92], who performed the adsorption of As species (As(III) and As(V)) on the HA-MNP surface, having little interference from iron in the competition for the NP binding sites. These results demonstrated that As species can be efficiently removed using functionalized Fe-oxide NPs (nanohybrid or composite materials) from natural water even in the presence of interfering adsorption ions.

### 4.2. Pb(II) Adsorption Experiments

#### 4.2.1. pH and Adsorption Mechanism of Pb(II)

**Figure 5:**
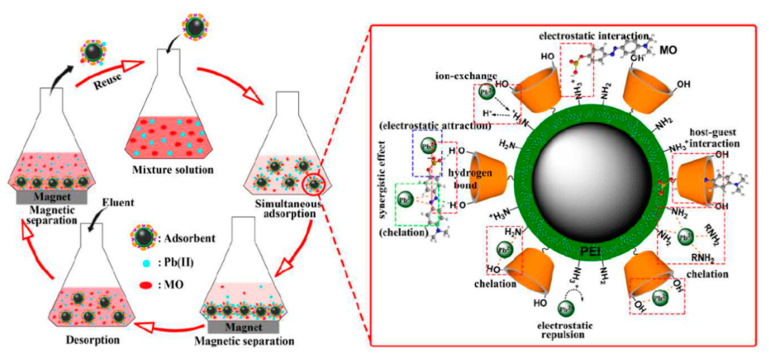
A versatile β-CD and PEI bi-functionalized magnetic nanoadsorbent with spatially separated sorption sites (denoted as Fe_3_O_4_-PEI/β-CD) was successfully constructed for simultaneous removal of methyl orange (MO) and Pb(II) from complex wastewater through multiple mechanisms (such as electrostatic attraction, host–guest inclusion, chelating, etc.); reproduced with permission of Elsevier [114].

As we already discussed for As, the adsorption performance of an adsorbent is strongly affected by many factors, such as the temperature, pH, the type and size of the pores, the functional groups of the adsorbent surface, the type of adsorbate/adsorbent interaction, and, mainly, the nature of the adsorbate [106,107,108,109,110,111,112,113]. There are various mechanisms such as electrostatic attraction, host–host inclusion, chelation, etc., that are involved in the adsorption process [30,114]. For example, Nejad et al. [113] demonstrated that the pH is an important factor that cannot be overlooked in the heavy metal ion adsorbing process. The main reason is that it affects the surface charge of adsorbents, the ionization state of the adsorbates, binding sites of the Fe-oxide NPs, the degree of the surface area of ionization charge, the speciation of the adsorbate, and surface complexation [113,114,115,116,117,118,119,120]. Based on these factors, a proper pH value for the Pb ion adsorption process must be previously determined. Specifically, for a pH range of 2.0–6.0, Pb species exist exclusively as Pb(II) ions in the solution, being these the predominant adsorbing form of Pb(II). For a pH higher than 6.0, Pb ions undergo via hydrolysis to Pb(OH)^+^ and Pb(OH)_2_ precipitates [108,115,118], which are electrostatically unfavorable for the adsorption reaction [113,114,117]. Another factor that can strongly affect the Pb(II) uptake is the concentration of hydrogen (H^+^) or hydronium (H_3_O^+^) ions that increase their fractions with the decrease in the pH. Consequently, it coexists in a competitive adsorption process that may occur between H^+^ or H_3_O^+^ ions and Pb(II). On increasing the pH, the competitive effect of H_3_O^+^ decreases, favoring the uptake process of Pb(II) ions on the free binding sites [106,115]. The p.z.c. of the adsorbent can reflect the influence of the pH on the adsorption process [116]. Thus, the measurements of pH_p.z.c._ should also be considered for the pH-sensitive adsorbent. The pH_p.z.c._ is the value of pH in which the solution possesses equal numbers of positive and negative charges. On the one hand, at values below pH_p.z.c._, the surface is positively charged, and the lower the pH is, the more positive is the surface charge of the adsorbent. In the last condition, the adsorption would be difficult due to the charge repulsion. On the other hand, at pH values higher than pH_p.z.c._, the surface of the material is negatively charged, favoring, in this case, the adsorption of metal ions [106,107,113,114,116]. Nejad et al. [113] reported that for a pH_p.z.c._ = 8.8, the highest adsorption capacity of the ${\mathrm{Fe}}_{3}O_{4}$-ETT for Pb(II) was observed at pH 5.0. This result indicated that the ${\mathrm{Fe}}_{3}O_{4}$-ETT surface is positively charged, suggesting that there is no electrostatic interaction between ${\mathrm{Fe}}_{3}O_{4}$-ETT and Pb(II). However, Pb(II) ions, as found in a Lewis acid, can interact with the π-electron pairs of the carbonyl functional group (Lewis’s base) on the triazinetrione ring of ${\mathrm{Fe}}_{3}O_{4}$-ETT [113]. In the same manner, the adsorption of the Pb(II) for ${\mathrm{Fe}}_{3}O_{4}$@−PEI/β−CD occurred to pH < pH_p.z.c._. Therefore, the adsorption capacities can be explained by the fact that the amino groups and oxygen-containing groups exhibit a strong chelating ability for Pb (II) ions [114]. However, the highest adsorption capacity for Pb(II) was found in pH = 5.0. This result indicates that the ${\mathrm{Fe}}_{3}O_{4}$-ETT surface is positively charged, suggesting that there is no electrostatic interaction between ${\mathrm{Fe}}_{3}O_{4}$-ETT and Pb(II). In particular, the nitrogen and oxygen-containing functional groups (with a strong chelating ability on the as-used adsorbent) played a crucial role in the complexing of Pb(II) [114]. A different adsorption mechanism is presented by Guo et al. [116], where the pH_p.z.c._ of ${\mathrm{Fe}}_{3}O_{4}$_-g_$-C_{3}N_{4}$ nanohybrids was found to be around 3.4, but an adsorption the capacity quickly improved with the pH increasing from 4 to 6 [116]. In this case, the adsorption was given by the electrostatic attractions, although the chemical interaction played a major role in adsorbing metal ions [116]. Figure 5 summarizes the main mechanisms of Pb(II) adsorption using Fe-oxide NPs.

Briefly, it could be inferred that the dependence of the adsorption capacity on the pH value can be strongly attributed to the change of the surface chemistry of the adsorbents with a pH modification. For example, if the surface of the adsorbents presents carboxyl groups at pH < pH_p.z.c.,_ the adsorption becomes low. As the pH increases, the carboxyl groups convert into anions carboxylate and adsorption gradually increase until pH > pH_p.z.c_. Hence, carboxyl groups completely turn into carboxylate anions with almost no change in the adsorption [118]. When the surface of the adsorbent presents hydroxyl groups (e.g., graphene) at a low pH, the number of H^+^ ions increases and the -OH groups become -${\mathrm{OH}}_{2}^{+}$. This last chemical reaction leads to the adsorption capacity of Pb(II) ions on the surface of the adsorbent to decrease. At a high pH, the -OH groups are ionized to -O^−^; thus, increasing the adsorption of Pb(II) ions [119]. Zhu et al. [110] reported the change of the ζ-potential with the pH of Fe_3_O_4_ NPs, which is positive (+28 mV) due to the formation of + Fe-OH_2_ in a basic environment. However, when it is functionalized to obtain L-Cyst-Fe_3_O_4_ NPs, the composite has a negative zeta potential (−30.2 mV). Therefore, it is worth mentioning that the L-cyst is negatively charged due to the carboxyl group present for pH < pH_p.z.c._ (5.1), but it is positively charged for pH > pH_p.z.c._ (5.1) due to the presence of the ammonium groups. Consequently, at pH = 6.0, L-Cyst-Fe_3_O_4_ NPs have shown a negative zeta potential and an efficient Pb(II) removal [110].

Finally, the adsorption capacity of the adsorbent for metallic iron not only depends on the hydrolysis capacity of the metal ions and the competitive adsorption of coexisting materials in the aqueous solution, but it is also influenced by the chemical and physical properties of the adsorption. Thus, the adsorption capacity presents a gradually increasing trend when the pH value increases. This is attributed primarily to two main reasons: (i) the greater competition of metallic iron than H^+^ for the combination of organic groups in acidic solutions which can cause an increase in adsorption, and (ii) the fact that metals have the tendency to hydrate to form OH^−^ groups with pH increases. Thus, they present a more effective size and greater mobility [112,118].

#### 4.2.2. Effect of Initial Concentration on the Uptake of Pb(II)

Jia et al. [115] reported that a high initial concentration of Pb(II) ions provided a higher driving force between the solid–liquid interfaces to overcome resistance during the mass transfer process, resulting in a higher adsorption rate, until the initial concentration of Pb(II) ions reaches its equilibrium condition, which can also be affected in the presence of other divalent metals [121]. However, the percentage of Pb(II) removal decreases with increasing concentrations of Pb (II). This may be due to the occupation of available adsorption sites. In other words, they are fulfilled with high concentrations of Pb(II), preventing the capture of more ions [30,114,121].

#### 4.2.3. Effect of Dosage on Pb(II) Removal

An increase in the adsorbent dosage may result in the presence of excessive adsorption sites and the aggregation of the adsorbents in the solution. Therefore, causing a reduction in the effective available adsorption sites for the removal of Pb(II) and also in the adsorption capacity [115]. As an example, we took the ${\mathrm{Fe}}_{3}O_{4}$-ETT hybrid that showed an improvement of the adsorption capacity when adsorbent doses increased from 1.0 × 10^−6^ to 2.0 × 10^−5^ kg for Pb(II), and then decreased gently on increasing adsorbent amounts. Nevertheless, the adsorption capacities after using 2.0 × 10^−5^ kg decreased significantly. This results indicated that the active adsorption sites were effectively reduced for the Pb(II) adsorption with the subsequent increase in the ${\mathrm{Fe}}_{3}O_{4}$-ETT dosage, showing the agglomeration of ${\mathrm{Fe}}_{3}O_{4}$-ETT NPs [113]. Thus, it can be inferred that with the change of the adsorbent dose, there is a change in the removal efficiency percentage. The adsorption capacity for Pb(II) rises with an increasing ion concentration until reaching the maximum adsorption capacity [117]; compared to other adsorbents, they show a quick adsorption rate [118].

#### 4.2.4. Temperature Dependence of the Pb(II) Removal

Pb(II) adsorption improves with an increasing temperature, suggesting that the process has an endothermic nature. Especially, it is assumed to be due to increased diffusion, the increased surface area of the adsorbent, and decreased viscosity of the solution. Therefore, by increasing the number of adsorption sites, due to the breaking of some internal bonds located on the edge of the particles, the adsorption of Pb(II) from the aqueous solution increases [106,119].

It was found that the uptake of Pb(II) by ${\mathrm{Fe}}_{3}O_{4}$@−PEI/β−CD was highly dependent on temperature and initial concentrations of adsorbates. High temperatures are more beneficial for the adsorption removal. For Pb(II) ions, the uptake amount increased gradually as initial concentration increased [107,114]. On the other hand, the results also suggested that when the temperature increased, the nanocomposites showed a tendency to lose weight that may be due to the elimination of residues [121]. Thus, it should be important to mention that thermal stability is another important factor that affects the direct application of an adsorbent. Therefore, under a high-temperature environment, the adsorbent must work properly, i.e., the composite degradation should be avoided. In general, it should be mentioned that nanocomposites have also shown a tendency to lose weight associated with ion removal [122]. A good example of composite material is the mHAP-oMWCNTs, which has a good thermal stability and achieves a mass balance at 600 °C, where the mass loss is less than 14% up to 1000 °C. The weight loss at high temperatures in this composite might have resulted from the removal of oxygen-containing groups [123]. 

#### 4.2.5. Simultaneous Removal of Divalent Metal Ions

The competitive adsorption of coexisting ions to the binding sites is often a serious problem when using conventional adsorbents for heavy metal removal. However, the magnetic adsorbents not only have the hole as a mesopore structure with a large surface area and pore volume, but also their ferromagnetic features. These characteristics make them effective and convenient adsorbents for heavy metals removal if magnetic remediation is the main process [121].

Jia et al. [115] studied the adsorption of Pb(II) in the presence of coexisting Cd (II), Ni (II), Cu (II), Pb(II), and Zn (II) ions on ${\mathrm{Fe}}_{3}O_{4}$@$\mathrm{Si}0_{2}@\mathrm{PEI}–\mathrm{NTDA}$, resulting in a higher removal efficiency for Pb(II) than that for other metal ions. The competitive adsorption performance was given in the following order: Pb(II) > Cd(II) > Zn (II) > Cu(II) > Ni(II). According to Pearson’s hard–soft acid–base theory, Pb(II) can be classified as a borderline acid and prefers bonding to ligands containing N-donor atoms. ${\mathrm{Fe}}_{3}0_{4}$@$\mathrm{Si}0_{2}@\mathrm{PEI}-\mathrm{NTDA}$, with a large number of N atoms in the polymer resin, may coordinate to Pb(II) rather than to Cd(II), which is classified as a soft acid. This behavior was also observed using ${\mathrm{Fe}}_{3}O_{4}$-FeMo$S_{4}$-MgAl-LDH, which stemmed from the soft Lewis base nature of sulfide Mo$S_{4}^{2+}$ ions [121]. They observed that the heavy metal ions acted as the soft Lewis acid and could be rapidly recovered, and selectivity captured from the aqueous solutions. The heavy metal ions adsorbed followed the sequence of Pb(II) > Cd (II) > Cu(II) [121].

#### 4.2.6. Simultaneous Pb(II) and Organic Pollutants Adsorption

Organic dyes and heavy metals had been widely found to coexist in groundwater or wastewater [114]. The uptake amount of Pb(II) increases significantly with an increasing concentration of co-existing MO. This synergetic effect could be since anionic MO adsorbed on the ${\mathrm{Fe}}_{3}O_{4}$@–PEI/β–CD surface would provide additional active sites (such as N-containing groups and sulfate groups) for Pb (II) uptake. The cationic Pb(II) seems to interact with -SO^3−^ of MO via electrostatic attraction; thus, improving the adsorption amounts of Pb (II) [114]. Wang et al. [111] reported the adsorption of Pb(II) and methylene blue (MB) on mHAP-oMWCNTs. The authors considered that the adsorption of Pb(II) is given by the ion exchange effect of HAP due to the content of Ca(II). For MB, the ion exchange effect of HAP no longer worked well and, consequently, the adsorption process mostly depended on oxygenic functional groups (-COOH, -OH) [123].

#### 4.2.7. Removal of Pb(II) and Organic Compounds

From Table S3, it can be seen that the highest adsorption capacity of 285.3 mg g^−1^ was obtained for nano-${\mathrm{Fe}}_{3}O_{4}$@$\mathrm{Si}0_{2}@\mathrm{PEI}–\mathrm{NTDA}$ [115]. It provides numerous amine and anhydride groups that improve the adsorption capacity of the adsorbent due to their chelating configuration. Thus, the nano-${\mathrm{Fe}}_{3}O_{4}$@$\mathrm{Si}0_{2}@\mathrm{PEI}–\mathrm{NTDA}$ nanohybrid joins the binding capability of amino groups and functionalized carboxyl groups in the outer PEI-NTDA layer, substantially enhancing the adsorption properties of nano$–{\mathrm{Fe}}_{3}O_{4}$. On the other hand, the saturation magnetization (*M_s_*) value decreased drastically to 21.6 emu g^−1^, but the material was still susceptible to applied magnetic fields. Regarding functionalized Fe-oxide NPs, we bring another good example of drastic magnetization reduction (*M_s_* value of 3.9 emu g^−1^) that was found in an ${\mathrm{Fe}}_{3}0_{4}@C@\mathrm{Ti}0_{2}$ nanotube composite (labeled as 3FeCTi). This nanocomposite was tested to Pb(II) removal, reaching an adsorption of 92% even in the presence of organic contaminant Rhodamine B (RhB). In other words, the magnetic remediation process showed that the low *M_s_* of the 3FeCTi nanohybrid does not affect the adsorption capacity and the 3FeCTi nanohybrid can first adsorb Pb (II) efficiently and still be separated from the effluent using an external magnetic field. A complex matrix formed by carbon nanotubes and ${\mathrm{TiO}}_{2}$ can be considered as a good candidate to prepare adsorbent materials applied to Pb(II) removal.

#### 4.2.8. Pb(II) Isotherm Models

Equilibrium isotherm models explain how metal ions are distributed in liquid/solid phases [107]. When the Freundlich model better fits the experimental data, it indicates that the surface of the nanocomposite is heterogeneous and the adsorption of Pb(II) occurs in multilayer adsorptions with more surface heterogeneity [11,113]. On the other hand, the Langmuir model is reasonable for a homogeneous adsorption process that takes place at the adsorbent surface, where there are no intermolecular interactions among the adsorbed molecules and all sites are identical and energetically equivalent for the adsorbate [115,119]. The heavy metal ions, captured by the adsorbent, mostly follow the pseudo-second order model [117,118,121]. The adsorption process is well described by this intraparticle diffusion model; thus, the rate of adsorption of heavy metals is mainly controlled by the diffusion rate within the material pores [117,118,122].

#### 4.2.9. Simultaneous Adsorption in Real Waters with Transition Metal-like Ions

The uptake of several divalent heavy metals, such as Ni(II), Pb(II), Cu(II), and Ni(II) from river water, was studied by Fato et al. [124] using mesoporous Fe_3_O_4_ NPs. The pH dependence of the divalent heavy metals was tested at 25 °C using an initial concentration of 10 mg L^−1^. The removal performance of 100% was achieved for a pH higher than 6.0 (a reduction in the removal efficiency is achieved below that pH). The adsorption mechanism was found to be electrostatic in nature. At around pH = 4.0, the zeta potential started to increase to –30 mV and fluctuated from –20 to –30 mV. This explains the higher uptake of the Fe-oxide NPs, since these values are recommendable for highly stable nanocolloids. The simultaneous removal was also tested in river water. The removal efficiency percentage varied from 60% to 80% for the Pb(II), Cd(II), and Cu(II) heavy metals in an equilibrium time of 120 min. In the case of Ni(II), a removal efficiency percentage of 40% was achieved for an equilibrium time of 160 min. No 100% removal efficiency was achieved for the simultaneous experiment, revealing the competitive adsorption behavior for the divalent heavy metal uptake.

### 4.3. Physicochemical Properties of Nano-Fe_3_O_4_ and Nano-γ-Fe_2_O_3_ Influencing As and Pb(II) Adsorption

Up to here, we discussed the influence of testing different physicochemical parameters in the As and Pb(II) adsorption processes using several Fe-oxide nanoadsorbents, and we can affirm that both Fe_3_O_4_ and γ-Fe_2_O_3_ NPs are excellent candidates to achieve a high percentage removal even in magnetic nanoarchitectures with small saturation magnetization, because the surface coating with inorganic and organic agents modifies the zeta potential and p.z.c. of the adsorbent and dotes the surface of chemical groups that favor the chemisorption of the heavy metals as shown in Figure 5, in case of Pb(II) adsorption; also discussed in Section 4.1.3 and Section 4.2.1. regarding the adsorption mechanisms. Moreover, in cases where nano-Fe_3_O_4_ and nano-γ-Fe_2_O_3_ were tested alone for heavy metal removal, an endothermic reaction took place as suggested by Liu et al. [22].

**Figure 6:**
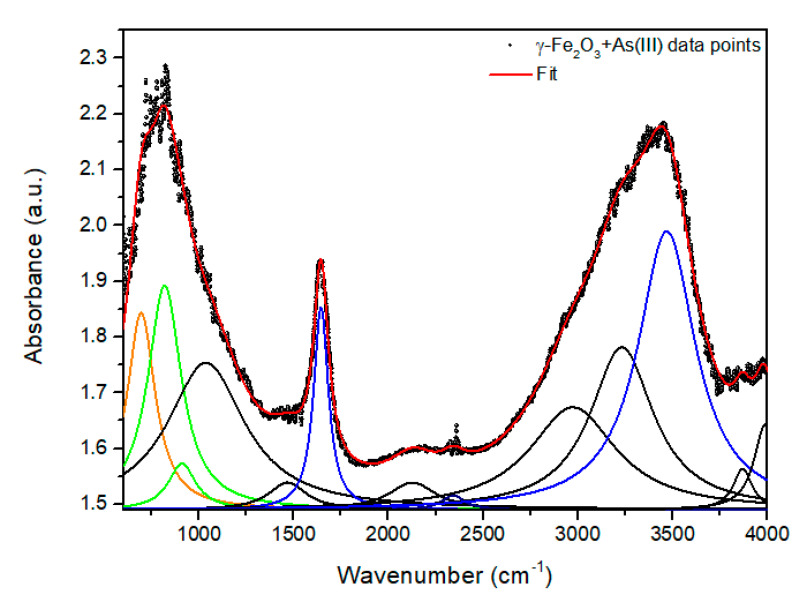
Fitted FTIR spectrum of the γ-Fe_2_O_3_ NPs in As(III) solution. In the graph, the orange color indicates the Fe-O stretching mode, the green color to As-O, the blue color to O-H, and the black represents the background contribution. Figure developed by the authors.

**Figure 7:**
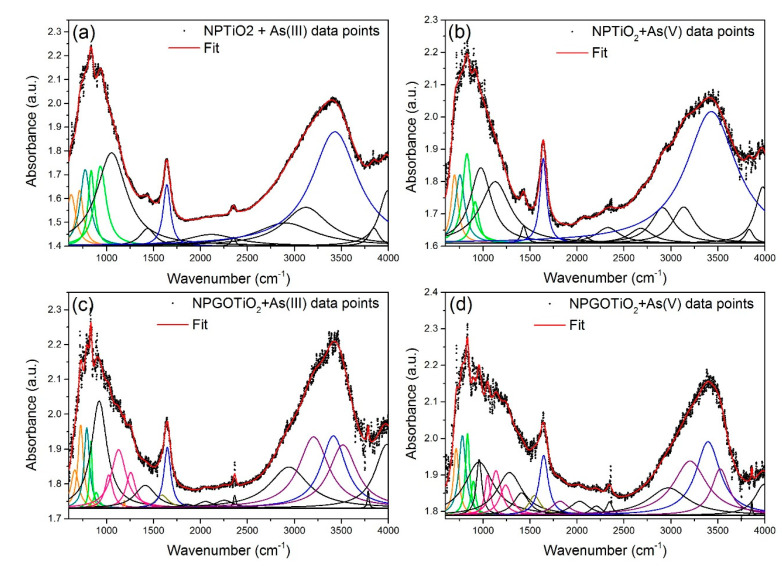
Fitted FTIR spectra of the NPTiO_2_ in (**a**) As (III) and (**b**) As (V) solutions, and NPGOTiO_2_ in (**c**) As(III) and (**d**) As(V) solutions. In the graphs, the orange color indicates the Fe-O stretching mode, the dark cyan is due to Ti-O, the green color to As-O, the pink color to C-O, the dark yellow to C=C, the blue color to O-H, the purple color to C-OH and COOH groups, and the black represents the background contribution. Figure developed by the authors.

On the other hand, a Fourier-transform infrared (FTIR) spectroscopy analysis was carried out in order to analyze the physicochemical properties of the functional IR groups of As(III) and As(V) species adsorbed onto bare γ-Fe_2_O_3_ NPs, binary NPTiO_2_, and ternary NPGOTiO_2_ nanocomposites, and also to confirm the adsorption mechanisms and physicochemical properties of the Fe-oxide NPs. A multiple peak fit was determined, where each noticeable IR mode was represented by a Lorentzian peak as presented in Figure 6 and Figure 7. Furthermore, the center positions of the Lorentzian lines are shown in Table 3. The characteristic IR broad bands of the As-O vibration mode have been previously identified at 800–950 cm^−1^ for As(V) [125,126]. In the case of As(III) adsorption, the As-O band shifted from its characteristic position (at 780–800 cm^−1^) to the same range of As(V). This is because of the As(III) reduction caused by γ-Fe_2_O_3_ NPs. Moreover, it seems that TiO_2_ contributed negatively to the shifting, since Pena et al. [125] mentioned that As-O groups weakened with As(III) adsorption onto TiO_2_ NPs.

According to Guivar et al. [42,43], γ-Fe_2_O_3_ characteristic absorption bands are located at 650–700 cm^−1^. In contrast to this, in Table 3, absorption bands are more slightly dispersed. This could be attributed to nano-γ-Fe_2_O_3_ tetrahedral sites [127] that interact with TiO_2_ NPs and As species. Additionally, it must be mentioned that nano-γ-Fe_2_O_3_ can present remaining peaks at 800–900 cm^−1^ [128,129], so part of the residual in this range in Figure 6 and Figure 7 could be due to these infrared (IR) absorption bands.

From these results, it can be inferred that the water functional group O-H is present in all samples, as a wide peak centered at 3300–3500 cm^−1^ (stretching vibration) and a thin peak ca. 1640 cm^−1^ (bending vibration). However, in the presence of graphene oxide, this peak overlaps due to the stretching vibration band of the C=C group (see Figure 7c,d) [42]. In presence of GO (see Figure 7c,d), carboxyl groups (C-O), alkoxy, epoxy, and carboxy have been identified at 1000–1300 cm^−1^ [42].

Despite several investigations being reported for the adsorption and removal of As species by using several magnetic nanohybrids, only few information has been found in the literature regarding the after-adsorption properties, especially explaining the surface mechanism for As removal. For example, Liu et al. [22] studied the As adsorption on Fe_3_O_4_ NPs from water; and Dutta et al. [21] proposed a surface complexation in a hollow polyanine microsphere/Fe_3_O_4_ nanocomposite. In both works [21,22], the As removal was explained based on the redox reaction and As–Fe complex via the ligand exchange mechanism. On the other hand, in a previous work, we were able to show that all studied samples were composed by pure γ-Fe_2_O_3_ NPs, as demonstrated by Mössbauer spectroscopy studies at 12 K [42]. Thus, the redox reaction mechanism, as suggested by Liu et al. [22], could not be expected because the core–shell configuration of Fe_3_O_4_/γ-Fe_2_O_3_ NPs is a requirement and a competitive adsorption behavior between Fe(II) and Fe(III) species is not favored in pure nano-γ-Fe_2_O_3_ samples. Here, it is important to point out that the adsorption applications must be carried out immediately after sample preparation in case of the co-precipitation method (this to have a redox reaction mechanism) since the surface will react quickly and the Fe_3_O_4_/γ-Fe_2_O_3_ core–shell configuration will be time dependent for sizes smaller than 10 nm as we discussed in this review and, hence, significantly affecting the As or Pb(II) adsorption processes.

Another interesting scientific report is that from Mikutta et al. [130], who investigated the ternary complex formation between arsenate and ferric iron complexes conjugated with HA by means of X-ray absorption spectroscopy. They found that 70% of As coordinated with Fe(III)-HA via an inner sphere complexation, forming a monodentate binuclear and monodentate mononuclear complex. It was also proposed that a complexation with Fe(III) behaves as an electron acceptor that increases the reduction in As(V); however, in the case of trivalent nano-γ-Fe_2_O_3_ samples, future studies are needed.

### 4.4. Regeneration and Reuse of Magnetic Nanoadsorbents

Once the Fe-oxide NPs show a high removal efficiency for heavy metals even in the presence of interferents and are applied in real water bodies, their regeneration and reuse must be studied to prove their sustainability. With the increase in synthesis and production of Fe-oxide NPs over the last 30 years, their release to the environment is inevitable and cycle regeneration must be carried out to control their liberation and reuse for diverse applications. Some works have reported the regeneration of magnetic NPs, for example, that of Shakeri et al. [131], who reported the regeneration protocol for the Fe_3_O_4_-based melamine-rich covalent organic polymer used for the removal of Auramine O (AO) and Rhodamine B (RB). The ethanol extraction technique was performed on the Fe-oxide NPs under nine cycles and the removal efficiency was 95% for AO and 88% for RB, suggesting the potential removal character of the Fe-oxide NPs. In another work, Behbahani et al. [121] tested the regeneration of Fe_3_O_4_/FeMoS_4_/MgAl-LDH using (i) 20 mL of 0.1 mol L^−1^ HCl to achieve a surface heavy metal desorption, (ii) washing with distilled water three times (final pH of 6.0) and, finally, (iii) drying at 60 °C for the next use. After six cycles, the removal percentage was reduced by 5.1% for Pb(II), 6.7% for Cd (II), and 8.5% for Cu(II) heavy metals. Xue et al. [132] studied the regeneration cycles for Fe_3_O_4_ NPs modified with HA, using 0.1 mol L^−1^ HCl as the regenerative agent. After four desorption cycles, the adsorbent showed 95% of removal efficiency for Pb(II), Cu(II), Cd(II), and of around 70% for Ni(II) in a high acidic medium of pH = 1.0. In addition, Ramos-Guivar et al. [133], using zeolite-type 5A functionalized γ-Fe_2_O_3_ NPs, demonstrated that a removal efficiency of 82% was achieved after seven regeneration cycles for 0.1 mol L^−1^ HCl, 70 mg L^−1^ Pb(II), and pH = 5.5. Despite all the previous magnetic nanoadsorbents depicting high removal efficiency after regeneration, the use of HCl at the pilot and industrial levels is still an open theme. Moreover, the physicochemical composition of the adsorbents needs to be deeply studied to corroborate the permanence of the structural and magnetic properties.

### 4.5. Cost Evaluation

There are few literatures that discuss the cost/prices and industrial-level implementation of these nanoadsorbents focusing on the water magnetic remediation method. A good example is given by Baig et al. [134], who discussed the prices of granulated nanoadsorbents. These authors suggested that the cost/price may vary between 1 and 5.14 USD/g of As removed for six different adsorbents, including Fe-oxide-coated sorbents, granular ferric hydroxide, iron ore, and zero valence iron. However, considering the case of functionalized Fe-oxide NPs (functionalization with inorganic and organic agents), it drastically increases their production costs. For example, if Fe-oxide NPs are combined with multiwall carbon nanotubes, these nanohybrids will require several steps to activate their surfaces with carboxyl and hydroxyl groups, which often take several hours. Thus, the use of acidic activators that, at industrial levels, leads to the contamination and abuse of several liters of water. The same procedure is required for the chemical activation of GO, limiting its use for industrial applications. The combined nanohybrids of two, three, or even four phases will increase the removal efficiency but will also rise the cost of production. Hence, the nanomaterials used for water magnetic remediation must have a high purity, but without sacrificing the removal efficiency. By evaluating the performance of a pure nano-γ-Fe_2_O_3_ at laboratory conditions [42], a removal efficiency of 85% for As(III) and As(V) was achieved, and the cost evaluation, regarding chemical reactive, water amounts, and pre-evaluation laboratory adsorption, suggested prices of 3.68–5.18 USD/g As removed, assuming an initial concentration of 136 mg L^−1^, which is higher than the one reported in Southern America countries (concentrations of 50 μg L^−1^) [17]. However, the long quantities to be used of these nanoadsorbents (in terms of kilograms or tons) in real polluted water, at high industrial levels, have not been evaluated up to the moment; thus, this issue is still lacking.

Therefore, the major tested experiments were performed in batch adsorption in simulated polluted water (synthetic effluents), and no discussions have been conducted in the major literature about the scalability of the proposed nanohybrids; it becomes a challenge to have a cost evaluation of the potential nanoadsorbent in which the final price will not sacrifice the applicability because the employment of combined adsorbent will certainly increase the production cost, including variables such as reactive prices, the synthesis time and equipment, plant design, consuming energy, training personal, among others. A great approach has been recently proposed by Augusto et al. [135], where an upscale design was performed for nanomagnetic particle production. Definitely, in the upcoming years, the demand for the use of these nanoadsorbents will increase as the heavy metal pollution areas spread and their impacts will drastically affect the environment (even more than today). Thus, an urgent call to the mining companies and Environmental Sciences Ministers of all countries to invest in these kinds of nanotechnologies is necessary. In other words, it is also mandatory to have more rigorous control and protection laws of the water bodies conjugated with the development of new nanoadsorption hybrid materials to keep the human impact at an acceptable level.

## 5. Conclusions

Water remediation for cleaning heavy metal ions (e.g., As, Pb, Cd, etc.) by employing magnetic nanohybrids is a hot and emerging topic that still requires more investigation as suggested in this review that compiled some of the most relevant papers reported in the literature. Despite adsorption having been suggested as a fast and efficient method to treat polluted waters with As and Pb cations, the magnetic properties of magnetic Fe-oxide NPs should be well understood to facilitate the entire removal procedure, i.e., magnetic structures of nanohybrids need to be comprehended because these nanoadsorbents should be magnetically manipulated using an applied field either in bare or with functionalized surface adsorption properties. Two of the most common Fe-oxide NPs suggested to be used in the magnetic remediation process are nanomagnetite and nanomaghemite, but they have different magnetic and catalytic properties and, therefore, still need further investigation. However, by proving the efficient removal and nanotoxicity properties of Fe-oxide NPs, the next steps to be considered are the scale up process and industrial level applications, considering their impacts in the environment and the final cost. Therefore, improvements in the synthesis methods still need to be undertaken together with an in-detail characterization of course, before liberating big quantities of these nanoadsorbents in soil and aquatic environments. In the present review, it was first summarized that the chemical methods (strong emphasis in co-precipitation and thermal decomposition methods) often used to synthesize magnetic hybrid nanoadsorbents based on Fe_3_O_4_ and γ-Fe_2_O_3_ NPs and their main differences, as studied by many physicochemical techniques (X-ray diffraction, zero and in-field Mössbauer spectroscopy, XPS, and synchrotron radiation techniques). From a deeper characterization of Fe-oxide NPs, we have demonstrated that high resolution experimental techniques are necessary, and the in-field Mössbauer technique seems to be the most appropriate experimental method to differentiate between Fe_3_O_4_ and γ-Fe_2_O_3_ NPs with sizes smaller than 10 nm. Using data from the literature, we also showed that the synthesized nano-Fe_3_O_4_ is sensitive to quick oxidation to nano-γ-Fe_2_O_3_ (entire particle or only its surface) that occurred a few hours immediately after the sample drying process. The partial oxidation effect can lead to a core–shell-like model (Fe_3_O_4_/γ-Fe_2_O_3_), where competitive surface magnetic effects should be well understood. For instance, the surface spin effect causes a reduction in the *M_s_* to values of about 3.9 emu g^−1^ of the nanoadsorbents. In other words, for a further understanding of the adsorption mechanism that happens on Fe_3_O_4_ and γ-Fe_2_O_3_ NP surfaces, high resolution spectroscopy techniques should be applied in order to bring information about magnetic properties and their catalytic properties during the divalent cation’s removal process. In particular, the removal of As and Pb from contaminated synthetic effluent was discussed by focusing on different physicochemical parameters. Zeta potential must be a mandatory technique to study not only the Fe-oxide NPs colloidal stability, but also to calculate the p.z.c. of the adsorbent at different pH values, helping to figure out the adsorption mechanism. Moreover, the use of FTIR, XPS, in-field Mössbauer, and synchrotron radiation techniques (all together) will help to clarify the different adsorption and also functionalization mechanisms.

## 6. Future Perspectives

Regeneration and recycling properties suggest that the magnetic nanohybrids can be stored and reused for various adsorption–desorption tests. However, the protocols to desorb the heavy metals still require the use of acidic treatments and represent a future challenge in the field of magnetic nanoadsorbents. On the other hand, there is no reports bringing information on how to store and recover the heavy metal/acid complex. The magnetic nanohybrids can be used in environments with many coexisting anions, divalent metals, and organic compounds to simultaneously adsorb the heavy metals. However, there is also a lack in the literature regarding pilot and industrial applications of these functionalized (or not) Fe-oxide NPs, especially by mining companies that are still using conventional and long-period treatment cleaning procedures. Additionally, no cost evaluation has been available frequently in the literature. The major studies focused and competed for the better adsorbent (batch laboratory tests) by using binary and ternary nanoadsorbents that can compromise the price and scale up demands in upcoming years, mainly for countries in development that have aqueous environments polluted with coexisting heavy metals and other organic species. Finally, the after adsorption properties also need to be carried out to better understand the physicochemical properties of the non-functionalized and functionalized Fe-oxide NPs and their influences on the As and Pb(II) removal mechanism. This will help to identify the sustainability and storage of the magnetic properties and re-use of the magnetic NPs.

## Figures and Tables

**Table 1:** Refined mean values of hyperfine parameters estimated from the in-field Mössbauer spectra recorded at 14 K under an 8 T external magnetic field applied parallel to the γ-ray beam for the NPEDTA1 (4.0 nm). In case of NPEDTA2 (7.6 nm) and NPEDTA3 (7.0 nm) samples were taken from [30]. A and B are related to the tetrahedral and octahedral sites of the γ-Fe_2_O_3_ phase. Notice that all hyperfine parameters here corresponded to a nano γ-Fe_2_O_3_ with a size smaller than 10 nm. F (%) denotes the atomic proportions for site A and B, respectively. e is the canted spin surface layer. <ϵ> is the quadrupolar shifting, B_eff_ is the total effective field, and < B_hf_ > is the mean hyperfine magnetic field.

**Sample**	**Site**	<δ>(mm/s) **± 0.02**	<2ϵ>(mm/s) **± 0.02**	**<B_eff_>** **(T)** **± 0.5**	<θ> (°) ± 5	<B_hf_> (T) ± 0.5	F (%) ± 1	e (nm) ± 0.05
NPEDTA1	A	0.18	−0.01	58.8	26	51.8	28	0.38
	B	0.29	0.01	46.2	42	52.4	57	0.89
	C	0.27	−0.00	54.2	56	50.2	15	1.37
NPEDTA2	A	0.36	−0.00	59.6	21	52.2	43	0.24
	B	0.52	0.02	46.2	28	53.3	57	0.42
NPEDTA3	A	0.37	−0.04	60.2	14	52.7	40	0.10
	B	0.52	0.00	46.0	22	53.5	60	0.24

**Table 2:** Hyperfine parameters obtained from Mössbauer technique for bulk and nano γ-Fe_2_O_3_/Fe_3_O_4_. RAA denotes the relative absorption area.

Bulk γ-Fe_2_O_3_	Nano-γ-Fe_2_O_3_	Bulk Fe_3_O_4_, nano-Fe_3_O_4_
At 14 K, it has perfect asymmetric sextets.	At RT, the sextets collapse to a doublet or singlet-superparamagnetic-like regime (size < 10 nm). At 14 K, the in-field Mössbauer measurements reveal two or three magnetic components depending on particle size. Broadenings can still be significant due to overbarrier fluctuations of smaller particles.	Bulk stoichiometric Fe_3_O_4_ depicts two characteristic sextets at RT, while the nano-Fe_3_O_4_ presents a collapse spectrum to a doublet or singlet. At 6 K, the spectrum is fitted with three components of tetrahedral Fe^3+^, octahedral Fe^3+^, and octahedral Fe^2+^ [7].
Static hyperfine magnetic fields.	At RT, fluctuating hyperfine magnetic fields are presented. At 14 K, superparamagnetic relaxation is negligible and two defined sextets are observed.	Hyperfine magnetic fields at RT [1,7]: $B_{\mathrm{hf},A}=48.6\;T$ $B_{\mathrm{hf},B}=45.5\;T$ Isomer shifts at RT K [1,7]: $\delta_{A}=0.26\;\mathrm{mm}/s$ $\delta_{B}=0.67\;\mathrm{mm}/s$
Hyperfine magnetic fields at 14 K [30]: **$B_{hf,A}=52.0\;T$** **$B_{hf,B}=53.1\;T$**	At RT, the appearance of the complex shapes with mixed components that depend on the particle size, anisotropy energies, blocking temperature distributions, and magnetic interactions are observed. At 14 K, if sizes are smaller than 10 nm, strong spin canting behavior occurs, and the hyperfine parameters slightly differ. For sizes bigger than 10 nm, the hyperfine parameters are equal to the bulk expected ones.	perfine magnetic fields at 140 K [35]: bulk Fe_3_O_4_ $B_{\mathrm{hf},A,\;B-{\mathrm{Fe}}^{3+}}=50.4\;T$ $B_{\mathrm{hf},B-{\mathrm{Fe}}^{2.5+}}=48.2\;T$ $\delta_{A,\;B-{\mathrm{Fe}}^{3+}}=0.27\;\mathrm{mm}/s$ $\delta_{B-{\mathrm{Fe}}^{2.5+}}=0.76\;\mathrm{mm}/s$ Hyperfine magnetic fields at 140 K [35]: 21 nm Fe_3_O_4_ $B_{\mathrm{hf},A,\;B-{\mathrm{Fe}}^{3+}}=50.6\;T$ $B_{\mathrm{hf},B-{\mathrm{Fe}}^{2.5+}}=46.7\;T$ $\delta_{A,\;B-{\mathrm{Fe}}^{3+}}=0.37\;\mathrm{mm}/s$ $\delta_{B-{\mathrm{Fe}}^{2.5+}}=0.65\;\mathrm{mm}/s$ Hyperfine magnetic fields at RT [7]: 5.3 nm Fe_3_O_4_$B_{\mathrm{hf},A}=40.9\;T$ $B_{\mathrm{hf},B}=38.2\;T$ Isomer shifts at RT [1,7]: $\delta_{A}=0.33\;\mathrm{mm}/s$ $\delta_{B}=0.46\;\mathrm{mm}/s$
Isomer shifts at 14 K [30]: **$\delta_{A}=0.36\;mm/s$** **$\delta_{B}=0.48\;mm/s$**	For fittings an average <δ> for each site must be considered. RAA for site A (37.5%) and site B (62.5%).	They showed lines due to Fe^2^^+^ at about −3.0 and −0.5 mm/s. Not observed in resolved spectrum of nano-γ-Fe_2_O_3_ at 14 K.

**Table 3:** Position centers of main modes of vibration. This table was developed from data of the IR spectra shown in Figure 6 and Figure 7.

Samples and Chemical Groups		γ-Fe_2_O_3_ + As (III)	NPTiO_2_ + As (III)	NPTiO_2_ + As (V)	NPGOTiO_2_ + As (III)	NPGOTiO_2_ + As (V)
Fe-O		694	624.7	697	662.7	714
Ti-O		-	773	754	789	779
As-O		818.9	837.9	828.9	834	834.9
C-O (alcoxy)		-	-	-	1024	1048
C-O (epoxy)		-	-	-	1129	1137
C-O (carboxy)		-	-	-	1257	1240
C=C		-	-	-	1591	1548
H_2_O:	O-H	1644	1641	1641	1647	1645
	O-H	3469	3431	3430	3414	3395
C-OH		-	-	-	3205	3204

## Data Availability

The original data related to this research can be asked any time to the corresponding author’s email: juan.ramos5@unmsm.edu.pe.

## Supplementary Materials

The following are available online at https://www.mdpi.com/article/10.3390/nano11092310/s1. Table S1: structure and adsorption parameters of magnetic nanohybrids used for As(III) and As(V) adsorption, Table S2: the adsorption parameters for removal of arsenic and other pollutants from water using diverse magnetic nanohybrids, Table S3: lead, other metals, and pollutant adsorption parameters for magnetic nanohybrids. A list of acronyms was also included.
