# Supplementary material for: Differentiating Nanomaghemite and Nanomagnetite and Discussing Their Importance in Arsenic and Lead Removal from Contaminated Effluents: A Critical Review
Source: Nanomaterials (Basel). 2021 Sep 6;11(9):2310. doi: 10.3390/nano11092310 (PMC8471304; doi:10.3390/nano11092310)
Supplement: Supplementary file 1 [file nanomaterials-11-02310-s001.zip › nanomaterials-1362622-supplementary.pdf]

Supplementary Materials for

Differentiating Nanomaghemite and Nanomagnetite and Discussing Their Importance in Arsenic and Lead Removal from Contaminated Effluents: A Critical Review

Juan A. Ramos-Guivar [1,*], Diego A. Flores-Cano [1] and Edson Caetano Passamani [2]

[1] Grupo de Investigación de Nanotecnología Aplicada para Biorremediación Ambiental, Biomedicina y Agricultura (NANOTECH), Facultad de Ciencias Físicas, Universidad Nacional Mayor de San Marcos, Lima 15081, Venezuela; Perú; diego.flores4@unmsm.edu.pe
[2] Physics Department, Federal University of Espírito Santo, Vitória, ES 29075-910, Brazil; passamaniec@yahoo.com.br
* Correspondence: juan.ramos5@unmsm.edu.pe; Tel.: +51-1-914728212

List of acronyms

TABLE S1

γ-Fe_2O_3@starch	: starch functionalized nano-γ-Fe_2O_3.
MBC	: magnetized biochar (vegetal carbon) composite.
MtZ	: nano-Fe_3O_4-zeolite composite.
CMnano	: nano-γ-Fe_2O_3 plus concrete.
PVA – SPIONs	:polyvinyl alcohol-Superparamagnetic iron-oxide NPs (nano-Fe_3O_4 with possible oxidation towards nano-γ-Fe_2O_3, stabilized by PVA).
bilayer–OA@FeO NPs	: bilayer-oleic coated iron-oxide NPs (γ-Fe_2O_3 and Fe_3O_4).
FeO_x-GO-80	: amorphous iron-oxide-graphene oxide-80 wt% of amorphous iron-oxide.
iMNP	: magnetic NPs from iron-containing sludge. It mainly contained γ-Fe_2O_3.
GM	: graphene oxide supported Fe_3O_4.
γ-Fe_2O_3@CTF-1	: γ-Fe_2O_3/covalent triazine framework.
Fe_2O_3-ZrO_2/BC	: Fe_2O_3-zirconium oxide/black cumin.
BCA-Fe	: activated biochar covered with nano-Fe_3O_4.
HFe10k	: halloysite containing sample with iron-oxide particles in a 10% proportion and calcined.
BC-Fe	: bamboo Fe biochar, it contains Fe_3O_4.
γ-Fe_2O_3-TiO_2-GO	: γ-Fe_2O_3-titanium dioxide- graphene oxide.
MBP	: bio-based substances-stabilized Fe_3O_4 /γ-Fe_2O_3 after pyrolysis.
Fe_3O_4:S	: Sulfur doped nano-Fe_3O_4.
c-MNPs	: coated magnetic NPs (FeOOH coated γ-Fe_2O_3).
Fe_3O_4@CuO&GO	: Fe_3O_4/copper oxide and graphene oxide.
BMN nanocomposittes	: biomass-derived magnetic nanocomposite (γ-Fe_2O_3, α-Fe_2O_3 and zero valent iron).
20%FBC	: iron-impregnated biochar with 20% Fe to biomass ratio, it contains γ-Fe_2O_3 and α-Fe_2O_3.

TABLE S2

HA	: humic acid.
GO/Fe_3O_4/HA	: graphene/Fe_3O_4/humic acid.
HA-Fe_3O_4	: humic acid grafted Fe_3O_4.
NC-MA/L-Fe_3O_4	: nanocellulose-maleic anhydride/(ethylenediamine-ethylenediaminetetraacetic acid-ethylenediamine)- Fe_3O_4.
Fe_3O_4/NOG	: Fe_3O_4/non-oxidative graphene.
Fe_3O_4@sand	: Fe_3O_4 coated sand.
PAN/GO/γ-Fe_3O_4	: polyacrylonitrile/graphene oxide/γ-Fe_2O_3.

TABLE S3

$\gamma - \mathrm{Fe_20_3@SiO_2}$	: γ-Fe_2O_3 NPs functionalized with silica.
$\gamma - \mathrm{Fe_20_3} - \mathrm{SBA15}$	: γ-Fe_2O_3 NPs functionalized with mesoporous silica.
$\gamma - \mathrm{Fe_20_3}@OA$	: γ-Fe_2O_3 NPs functionalized with oleic acid.
$\gamma - \mathrm{Fe_20_3}@LA$	: γ-Fe_2O_3 NPs functionalized with lauric acid.
$\gamma - \mathrm{Fe_20_3}@L\text{-}arg$	: γ-Fe_2O_3 NPs functionalized with L-arginine.
$\gamma - \mathrm{Fe_20_3@HAp}$	: γ-Fe_2O_3 NPs functionalized with hydroxyapatite.
$\gamma - \mathrm{Fe_20_3@MWCNTs}$	: γ-Fe_2O_3 NPs functionalized with carbon nanotubes.
$\gamma - \mathrm{Fe_20_3} - \mathrm{EDTA1}$	: γ-Fe_2O_3 NPs functionalized with ethylenediaminetetraacetic acid.

$\gamma - Fe_2O_3 - EDTA2$: γ-Fe_2O_3 NPs functionalized with ethylenediaminetetraacetic acid.
$\gamma - Fe_2O_3 - EDTA3$: γ-Fe_2O_3 NPs functionalized with ethylenediaminetetraacetic acid.
$L - Cyst - Fe_3O_4$: L-cysteine functionalized Fe_3O_4 NPs.
$Fe_3O_4 - NH_2$: amine functionalized Fe_3O_4 NPs.
Fe_3O_4@PDA : Fe_3O_4 NPs functionalized with polydopamine.
Fe_3O_4-ETT : Fe_3O_4-epoxy-triazinetrione (ETT).
Fe_3O_4-PEI/β-CD : Fe_3O_4- polyethyleneimine /β-cyclodextrin.
Fe_3O_4@Si02@PEI-NTDA : Fe_3O_4/Silicon dioxide/polyethylenimine-1,4,5,8-naphthalenetetracarboxylic-dianhydride.
Fe_3O_4-g- C_3N_4 : Fe_3O_4-graphitic carbon nitride.
Fe_3O_4-SO_3H : sulfonated magnetic NP adsorbents.
Fe_3O_4@APS : 3-aminopropyltriethoxysilane functionalized magnetic NPs.
Fe_3O_4@ APS@AA-co-CA : 3-aminopropyltriethoxysilane - acrylic acid and crotonic acid copolymer functionalized magnetic NPs. CNTs/ Fe_3O_4 : thiol-functionalized multiwall carbon nanotube/ Fe_3O_4 nanocomposites.
MPTS-CNTs/ Fe_3O_4 : 3-mercaptopropyltriethoxysilane (MPTS)-the as-synthesized thiol-functionalized CNTs/ Fe_3O_4.
Fe_3O_4@C@TiO_2 : γ-Fe_2O_3/carbon particles/titanium dioxide.
Fe_3O_4 -$FeMoS_4$-MgAl-LDH : Fe_3O_4- $FeMoS_4^{2-}$ magnesium-aluminum layered double hydroxide.
SH-mSi@ Fe_3O_4 : thiol-functionalized magnetic mesoporous silica material.
mHAP-oMWCNTs : magnetic hydroxyapatite-immobilized oxidized multi-walled carbon nanotubes.
GFMNPECABs : glycine-functionalized magnetic NPs-entrapped calcium alginate beads.
GFMNPS: Glycine functionalized magnetic NPs.
RT: room temperature.
p.z.c. point of zero charge.
qe or qm: Langmuir's maximum adsorption capacity (mg g^{-1}).
k_F: Freundlich adsorption constant (L mg^{-1}).
k_s. Sips constant (L $mg^{-1})^m$.
K_T: equilibrium binding constant (L mg^{-1}).
k_L: Langmuir adsorption constant (L mg^{-1}).
q_{mDR}: Dubinbin–Radushkevich's maximum adsorption capacity.
DLS: Dynamic light scaterring.
AFM: Atomic Force Microscopy.
SEM: Scanning Electron Microscopy.
SAXS: Small-angle X-ray scattering.
EDS: Electron Dispersive X-ray Spectroscopy.
FESEM: Field Emission Scanning Electron Microscope.
BET: Brunauer-Emmett-Teller.

Table S1. Structure and adsorption parameters of magnetic nanohybrids used for As(III) and As(V) adsorption.

Adsorbent	Synthesis method	Size and shape	Surface area	Saturation magnetization (RT)	Adsor bates	Initial concentration (C_0) and equilibrium time (t_e)	Concentration range in isotherm experiments	Adsorption conditions	Kinetic and isothermal parameters	Ref.
γ-Fe_2O_3@starch	Coprecipitati on	TEM: 9.7 nm<br>XRD: 8.1 nm<br>spherical NPs	-	-	As(III)	C_0=1 mg L^{-1}	1.0-6.0 mg L^{-1}	T=27 °C<br>pH=7<br>dose=1 g L^{-1}	**Pseudo-second order:**<br>q_e=1.02 mg g^{-1}<br>k_2=11.55 g mg^{-1} min^{-1}<br>R^2=0.98<br>**Langmuir:**<br>q_m=8.6 mg g^{-1}<br>k_L=9.1 L mg^{-1}<br>R^2=0.98<br>**Freundlich:**<br>k_F=16.5 mg g^{-1} (L $mg^{-1})^{1/n}$<br>n=1.60<br>R^2=0.98<br>**Removal:**<br>99 %	[58]
MBC	Coprecipitati on	STEM: 18.1 nm (Fe_3O_4) clustered or aggregated on the surface (2-7 μm)	320.1 m^2 g^{-1}	-	As(III)	C_0=10 mg L^{-1}<br>t_e=1 - 1.5 h	1-12 mg L^{-1}	T=25 °C<br>pH=7<br>dose=2 g L^{-1}	**Pseudo-second order:**<br>q_e=3.75 mg g^{-1}<br>k_2=0.319 g mg^{-1} min^{-1}<br>R^2=0.9999<br>**Sips:**<br>q_s=5.49 mg g^{-1}<br>k_s=1.14 L mg^{-1}<br>R^2=0.9889<br>**Langmuir:**<br>q_m=5.06 mg g^{-1}<br>k_L=0.96 - 0.22 L mg^{-1}<br>R^2<0.9889<br>**Removal:**<br>68.4 %	[59]
MtZ	Alkaline precipitation and coating	SEM: 77 nm Fe_3O_4	38 m^2 g^{-1}	-	As(V)	C_0=100 mg L^{-1}<br>t_e=3 h	1-450 mg L^{-1}	T=25 °C<br>pH=5.5<br>dose=10 g L^{-1}	**Pseudo-second order:**<br>q_e=1.35 mg g^{-1}<br>k_2=0.109 g mg^{-1} min^{-1}<br>R^2=0.904<br>**Langmuir:**<br>q_m=6.211 mg g^{-1}<br>k_L=0.009 L mg^{-1}<br>R^2=0.995<br>**Freundlich:**	[60]

									k_F=0.209 mg g^{-1}<br>n=1.877<br>R^2=0.957	
CMnano	Coprecipitati<br>on	SEM: ~20 nm<br>XRD: 17 nm<br>round shaped	-	3.45 emu g^{-1}	As(V)	C_0=12.5 mgL^{-1}<br>t_e= 72 h	0.1-1300 mg L^{-1}	T=30 °C<br>pH=5<br>dose=20 g L^{-1}	**Pseudo-second order:**<br>q_e=0.618 mg g^{-1}<br>k_2=0.436 g mg^{-1} h^{-1}<br>R^2=0.991<br>**Langmuir:**<br>q_m=11.12 mg g^{-1}<br>R^2=0.995<br>**Gunary:**<br>q_m=8.82 mg g^{-1}<br>R^2=0.998<br>**Removal:**<br>85 %	[61]
PVA - SPIONs	Coprecipitati<br>on	TEM: 9 nm<br>(SPIONs),<br>SEM:9.5 nm<br>(SPIONs),<br>XRD: 10 nm<br>(SPIONs),<br>a=8.367 Å (SPIONs)<br>a=8.38 Å (SPIONs in<br>membrane)	-	74 emu g^{-1}	As(V)	C_0=0.86 mg L^{-1}<br>t_e=6 h	0.15-1.1 mg L^{-1}	T=25 °C<br>pH=3<br>dose=8.4 mg<br>L^{-1}	**Langmuir:**<br>q_m=52 mg g^{-1}<br>k_L=0.496 L mg^{-1}<br>R^2=0.991<br>**Freundlich:**<br>k_F=0.03 mg g^{-1}<br>n=0.94<br>R^2=0.985	[44]
bilayer–<br>OA@FeO NPs	Coprecipitati<br>on	SEM: 20-40 nm	6.53 m^2<br>g^{-1}	-	As(V)	C_0=0.03 mg L^{-1}<br>t_e= 2 h	0.001-0.15 mg L^{-1}	T=25 °C<br>pH=7<br>dose=1 g L^{-1}	**Pseudo-second order:**<br>q_e=11.9 μg g^{-1}<br>k_2=2 g mg^{-1} min^{-1}<br>R^2=0.968<br>**Langmuir:**<br>q_m=0.0328 mg g^{-1}<br>k_L=0.72 L g^{-1}<br>R^2=0.93<br>**Freundlich:**<br>k_F=1.8 L mg^{-1}<br>n=1.82<br>R^2=0.99	[63]
FeO_x-GO-80	Coprecipitati<br>on	TEM: 5 nm<br>amorphous	341 m^2<br>g^{-1}	-	As(III)	C_0=400 mg L^{-1}<br>t_e= ~250 min	25-1200 mg L^{-1}	T=23 °C<br>pH=7<br>dose=0.8 g L^{-1}	**Pseudo-second order:**<br>q_e=110 mg g^{-1}<br>k_2=0.001 g mg^{-1} min^{-1}<br>R^2=1.000<br>**Langmuir:**<br>q_m=147 mg g^{-1}<br>k_L=0.011 L mg^{-1}<br>R^2=0.991<br>**Removal:**	[64]

									>99.98 %	
					As(V)	C_0=300 mg L^{-1}<br>t_e= ~1450 min	25-350 mg L^{-1}	T=23 °C<br>pH=3<br>dose=0.8 g L^{-1}	**Pseudo-second order:**<br>q_e=114 mg g^{-1}<br>k_2=0.001 g mg^{-1} min^{-1}<br>R^2=1<br>**Langmuir:**<br>q_m=113 mg g^{-1}<br>k_L=0.295 L mg^{-1}<br>R^2=0.994<br>**Removal:**<br>>99.98 %	
iMNP	Coprecipitation	TEM: 14.3-45.1 nm<br>(avg.=23.5 nm)<br>quasi-spherical	145.5 m^2 g^{-1}	35.5 emu g^{-1}	As(V)	C_0=400 μg L^{-1}<br>t_e=60 min	1-10 mg L^{-1}	T=25 °C<br>pH=6.6<br>dose=0.2 g L^{-1}	**Pseudo-second order:**<br>q_e=1.879 mg g^{-1}<br>k_2=0.4753 g mg^{-1} min^{-1}<br>R^2=0.9996<br>**Langmuir:**<br>q_m=12.74 mg g^{-1}<br>k_L=6.025 L mg^{-1}<br>R^2=0.8976<br>**Freundlich:**<br>k_F=10.50 L g^{-1}<br>1/n=0.100<br>R^2=0.7236<br>**Removal:**<br>>90 %	[65]
GM	Coprecipitation	TEM: 5.5-12.5 nm<br>spherical,<br>agglomerated and<br>deposited on an<br>irregular shaped and<br>paper-like GO	-	-	As(III)	C_0=5 mg L^{-1}	-	T=22 °C<br>pH=9<br>dose=333 mg L^{-1}	As loading=23-29 mg g^{-1}<br>Removal >90 %	[66]
					As(V)	C_0=5 mg L^{-1}	-	T=22 °C<br>pH=3<br>dose=333 mg L^{-1}	As loading=10.4-41.7 mg g^{-1}<br>Removal >90 %	
γ-Fe_2O_3@CTF-1	Coprecipitation	STEM: 5-7 nm	1049 m^2 g^{-1}	-	As(III)	C_0=0.1 mg L^{-1}		T=25 °C<br>pH=7<br>dose=4 g L^{-1}	**Pseudo-second order:**<br>q_e=2.554 mg g^{-1}<br>k_2=-2.688 mg min g^{-1}<br>R^2=0.9999<br>**Langmuir:**<br>q_m=198.0 mg g^{-1}<br>**Removal:**<br>Maximum removal efficiency=97.1 %	[67]
					As(V)	C_0=0.1 mg L^{-1}<br>t_e<24 h (few minutes)			**Pseudo-second order:**<br>q_e=1.859 mg g^{-1}<br>k_2^{-1}=-3.709 mg min g^{-1}<br>R^2=0.9999	

									**Langmuir:**<br>q_m=102.3 mg g^{-1}<br>**Removal:**<br>96.6 %	
Fe_2O_3-ZrO_2/BC	Coprecipitation	TEM: 17-22 nm<br>XRD: 54nm<br>irregular shape	-	-	As(III)	C_0=1.0 mg L^{-1}<br>t_e=~2 h	0-0.12 mg L^{-1}	T=27 °C<br>dose= 2 g L^{-1}	**Pseudo-second order:**<br>q_e=0.4586 mg g^{-1}<br>k_2=45.0 g mg^{-1} min^{-1}<br>R^2=1<br>**Langmuir:**<br>q_m=1.01 mg g^{-1}<br>k_L=6.48 L mg^{-1}<br>R^2=0.97<br>**Freundlich:**<br>k_F=1.98 mg^{-1} $(Lmg^{-1})^{1/n}$<br>n=1.433<br>R^2=0.99<br>**Sips:**<br>q_s=0.99 mg g^{-1}<br>k_s=0.996 L mg^{-1}<br>R^2=0.98<br>**Redlich-Peterson:**<br>k_r=2.897 L mg^{-1}<br>R^2=0.99<br>**Removal:**<br>90 %	[68]
BCA-Fe	Coprecipitation<br>(BCA is synthetized by another method)	TEM-EDS: 10-25 nm for Fe_3O_4<br>porous	482.4 m^2 g^{-1}	-	As(V)	C_0=10 mg L^{-1}<br>t_e=1 h	2.5-1000 mg L^{-1}	T=25 °C<br>pH=7<br>dose=2 g L^{-1}	**Pseudo-second order:**<br>q_e=11.36 mg g^{-1}<br>k_2=0.02 g mg^{-1} min^{-1}<br>R^2=0.99<br>**Langmuir:**<br>q_m=85 mg g^{-1}<br>**Removal:**<br>86 %	[69]
HFe10k	Coprecipitation and calcination	XRD: 20 nm (nanoγ-Fe_2O_3)<br>spherical in ~50 nm clusters	65.4 m^2 g^{-1}	-	As(V)	-	0.01-25 mmol L^{-1}	T=22 °C<br>pH=5<br>dose=20 gL^{-1}	**Langmuir:**<br>q_m=178.5 mmol kg^{-1}<br>R^2>0.99	[70]
Fe_3O_4	Coprecipitation	DLS: 248 nm	35 m^2 g^{-1}	-	As(III)	C_0=2 mg L^{-1}<br>t_e=~5 h	2-400 mg L^{-1}	T=27 °C pH=7<br>dose=4 g L^{-1}	**Freundlich:**<br>k_F=1.79 mg g^{-1}<br>n=2.05	[71]
					As(V)	C_0=2 mg L^{-1}<br>t_e=~5 h	2-400 mg L^{-1}	T=27 °C pH=7<br>dose=4 g L^{-1}	**Freundlich:**<br>k_F=3.38 mg g^{-1}<br>n=1.5	
BC-Fe	Coprecipitation	-	28.9 m^2 g^{-1}	-	As(V)	t_e=2 h	0.1-1 mg L^{-1}	T=25 °C<br>pH=7	**Langmuir:**<br>q_m =90 mg g^{-1}	[72]

γ-Fe_2O_3-TiO_2-GO	Coprecipitation	TEM: 9 nm (γ-Fe_2O_3)<br>spherical γ-Fe_2O_3<br>Agglomerate TiO_2 (50 nm) and well defined using GO<br>XRD: 9 nm<br>a=8.3789 Å for γ-Fe_2O_3<br>a=3.7867 Å and b=9.4393 Å for TiO_2	82 m^2 g^{-1}	60 emu g^{-1}	As(III)	C_0=33 mg L^{-1}<br>t_e=20 h	1-400 mg L^{-1}	T=25 °C<br>pH=6.5<br>dose=0.56 g L^{-1}	**Pseudo-second order:**<br>q_e=25.3 mg g^{-1}<br>k_2=0.0013 g mg^{-1} min^{-1}<br>R^2=0.9998<br>**Langmuir:**<br>q_m=110.4 mg g^{-1}<br>k_L=0.06 L mg^{-1}<br>R^2=0.986<br>**Freundlich:**<br>k_F=13.7 mg g^{-1}<br>n=2.27<br>R^2=0.949<br>**Sips:**<br>q_s=121.1 mg g^{-1}<br>k_s=0.07 (L $mg^{-1})^m$<br>m_S=0.88<br>R^2=0.984<br>**Redlich-Peterson:**<br>A=7.9 L g^{-1}<br>B=0.14 L mg^{-1}<br>R^2=0.983<br>**Removal:**<br>73 %	[42]
					As(V)	C_0=33 mg L^{-1}<br>t_e=20 h	1-400 mg L^{-1}	T=25 °C<br>pH=6.5<br>dose=0.56 g L^{-1}	**Langmuir:**<br>q_m=127.2 mg g^{-1}<br>k_L=0.13 L mg^{-1}<br>R^2=0.997<br>**Freundlich:**<br>k_F=45.8 mg g^{-1}<br>n=5.39<br>R^2=0.93<br>**Sips:**<br>q_s=130.1 mg g^{-1}<br>k_s=0.159 (L $mg^{-1})^m$<br>m=0.88<br>R^2=0.998<br>**Redlich-Peterson:**<br>A=21.1 L g^{-1}<br>B=0.217 L mg^{-1}<br>R^2=0.996<br>**Removal:**<br>85 %	
MBP	Coprecipitation	TEM: 10 nm	-	45 emu g^{-1}	As(III)	-	5-300	T=25 °C<br>pH=6<br>dose=2 g L^{-1}	**Langmuir:**<br>q_m=17.2 mg g^{-1}<br>k_L=0.4 L mg^{-1}<br>R^2=0.954<br>**Freundlich:**<br>k_F=7.4 mg g^{-1}	[73]

									$n=5.7$<br>$R^2=0.843$	
					As(V)	-	5-300	T=25 °C<br>pH=6<br>dose=2 g L^{-1}	**Langmuir:**<br>$q_m=43.9$ mg g^{-1}<br>$k_L=1.2$ L mg^{-1}<br>$R^2=0.832$<br>**Freundlich:**<br>$k_F=18.6$ mg g^{-1}<br>$n=4.8$<br>$R^2=0.900$	
Fe_3O_4	Chemical Precipitation	XRD: 84 nm<br>a=8.38523 Å	12 m^2 g^{-1}	-	As(V)	-	0.1-8	T=20 °C<br>pH=7<br>dose=1 g L^{-1}	**Langmuir:**<br>$q_m=1.386$ mg g^{-1}<br>$k_L=8.06$ L mg^{-1}<br>$R^2=0.9963$<br>**Freundlich:**<br>$k_F=1.045$ mg g^{-1}<br>$n=6.35$<br>$R^2=0.9867$	[46]
Fe_3O_4:S	Novel chemical synthesis method, like chemical precipitation	TEM: 4.34-13.46 nm (avg. 7.84 nm)	-	37.1 and 39.9 emu g^{-1} before and after adsorption	As(V)	$C_0=20$ mg L^{-1}<br>$t_e=24$ h	5-50 mg L^{-1}	T=30 °C<br>pH=6<br>dose=0.5 g L^{-1}	**Pseudo-second order:**<br>$q_e=42.44$ mg g^{-1}<br>$k_2=0.00193$ g mg^{-1} min^{-1}<br>$R^2=0.999$<br>**Langmuir:**<br>$q_m=58.38$ mg g^{-1}<br>$k_L=0.971$ L mg^{-1}<br>$R^2=0.990$	[75]
cellulose@Fe_2O_3	Coprecipitation	TEM: 5-100 nm<br>XRD: 61 nm<br>a=8.3515 Å	113 m^2 g^{-1}	13.2 emu g^{-1}	As(III)	-	0-~27 mg L^{-1}	T=25 °C<br>pH=7<br>dose=1 g L^{-1}	**Langmuir:**<br>$q_m=23.16$ mg g^{-1}<br>$k_L=0.48$ L mg^{-1}<br>$R^2=0.9959$<br>**Freundlich:**<br>$k_F= 9.64$ mg g^{-1}<br>$n= 3.78$<br>$R^2=0.9656$	[47]
					As(V)	-	0-~27 mg L^{-1}		**Langmuir:**<br>$q_m=32.11$ mg g^{-1}<br>$k_L=0.039$ L mg^{-1}<br>$R^2=0.9836$<br>**Freundlich:**<br>$k_F= 3.25$ mg g^{-1}<br>$n= 2.06$<br>$R^2=0.9961$	

MNPs (γ-Fe_2O_3)	Coprecipitation	TEM: 23.5 nm<br>XRD: 5.9 nm<br>irregular shaped	145.5 m^2 g^{-1}	35.5 emu g^{-1}	As(V)	C_0=0.4 mg L^{-1}<br>t_e=~1 h	0-10 mg L^{-1}	T=25 °C<br>pH=6.6<br>dose=0.2 g L^{-1}	**Langmuir:**<br>q_m=12.74 mg g^{-1}<br>k_L=6.03 L mg^{-1}<br>R^2=0.898<br>**Removal:**<br>95 %	[77]
c-MNPs	Coprecipitation and heterogeneous nucleation technique	TEM: 37.0 nm<br>XRD: 4.9 nm<br>irregular shaped	157.1 m^2 g^{-1}	36.4 emu g^{-1}	As(V)	C_0=0.4 mg L^{-1}<br>t_e=~1 h	0-15 mg L^{-1}		**Langmuir:**<br>q_m=26.05 mg g^{-1}<br>k_L=0.34 L mg^{-1}<br>R^2=0.901<br>**Removal:**<br>>95 %	
Fe_3O_4	Chemical precipitation	TEM: 5 nm<br>XRD: 5 nm	179 m^2 g^{-1}	40 emu g^{-1}	As(III)	-	0-70 mg L^{-1}	T=27 °C<br>pH=7<br>dose=60 mg L^{-1}	**Langmuir:**<br>q_m=46.06 mg g^{-1}<br>k_L=0.1686 L mg^{-1}<br>**Removal:**<br>Maximum removal=97.5 %	[78]
					As(V)	-	0-50 mg L^{-1}	T=27 °C<br>pH=7<br>dose=60 mg L^{-1}	**Langmuir:**<br>q_m=16.56 mg g^{-1}<br>k_L=1.42 L mg^{-1}<br>**Removal:**<br>97.5 %	
Fe_3O_4@CuO&GO	A modified precipitation method	TEM: 20 nm<br>spherical and packed	159.60 m^2 g^{-1}	30 emu g^{-1}	As(III)	C_0=15 mg L^{-1}<br>t_e=12 h	0-~80 mg L^{-1}	T=25 °C<br>pH=7<br>dose=120 mg L^{-1}	**Langmuir:**<br>q_m=70.36 mg g^{-1}	[79]
					As(V)	C_0=15 mg L^{-1}<br>t_e=12 h	0-~80 mg L^{-1}	T=25 °C<br>pH=7<br>dose=120 mg L^{-1}	**Langmuir:**<br>q_m=62.60 mg g^{-1}	
Starch-bridged Fe_3O_4	A modified coprecipitation method	TEM: 26.6 nm<br>spherical shape	-	-	As(V)	t_e=4 h	0-400 mg L^{-1}	T=21 °C<br>pH=5<br>dose=1.7 g L^{-1}	**Langmuir:**<br>q_m=248.0 mg g^{-1}<br>k_L=0.5 L mg^{-1}<br>**Removal:**<br>>98 %	[80]
γ-Fe_2O_3	Commercial	TEM: 18.4 nm	51 m^2 g^{-1}	71.7 emu g^{-1}		t_e=~20 h	1-11 mg L^{-1}	T=23 °C<br>pH=7<br>dose=250 mg L^{-1}	**Langmuir:**<br>q_m=5 mg g^{-1}	[81]
γ-Fe_2O_3	Sol-gel	TEM: 12.1 nm	90.4 m^2 g^{-1}	64.3 emu g^{-1}	As(V)			T=23 °C<br>pH=7<br>dose=100 mg	**Langmuir:**<br>q_m=11.1 mg g^{-1}	

								L^{-1}		
γ-Fe_2O_3	Mechanochemical	TEM: 3.8 nm	203.2 m^2 g^{-1}	19.6 emu g^{-1}				T=23 °C<br>pH=7<br>dose=60 mg L^{-1}	**Langmuir:**<br>q_m=20 mg g^{-1}	
Fe_3O_4-γ-Fe_2O_3 mixture	Electrical wire explosion (EWE)	TEM: 34 nm<br>a=8.3763 Å	12 m^2 g^{-1}	91.8 emu g^{-1}	As(III)	-	1-7 mg L^{-1}	T=25 °C<br>pH=6<br>dose=1 g L^{-1}	**Langmuir:**<br>q_m=2.9 mg g^{-1}	[82]
					As(V)	-	1-7 mg L^{-1}	T=25 °C<br>pH=6<br>dose=1 g L^{-}	**Langmuir:**<br>q_m=3.1 mg g^{-1}	
Mesoporous γ-Fe_2O_3	A described in the reference hydrothermal process	spherical shape	35.7 m^2 g^{-1}	16.6 emu g^{-1}	As(V)	-	0-80 mg L^{-1}	dose=0.1 g L^{-1}	**Langmuir:**<br>q_m=73.2 mg g^{-1}<br>k_L=0.39 L mg^{-1}	[83]
BMN nanocomposites	A one-step pyrolysis process	TEM: 20 nm	-	-	As(III)	C_0=10 mg L^{-1}<br>t_e=6 h	0.5-50 mg L^{-1}	T=25 °C<br>pH=7<br>dose=2 g L^{-1}	**Langmuir:**<br>q_m=16.23 mg g^{-1}<br>**Removal:**<br>90 %	[84]
20%FBC	Thermal pyrolysis	-	297.13 m^2 g^{-1}	67.2 emu g^{-1}	As(V)	C_0=10 mg L^{-1}<br>t_e=24 h	0.25-100 mg L^{-1}	T=25 °C<br>pH=7<br>dose=5 g L^{-1}	**Langmuir:**<br>q_m=6.8 mg g^{-1}<br>**Removal:**<br>86.48 %	[85]
Fe_3O_4@TiO_2 magnetic nanosheets	Sol-gel method and a hydrothermal assisted crystallization strategy	AFM: ~1.5 nm (thickness)<br>XRD: 9 nm nanosheets	89.4 m^2 g^{-1}	20 emu g^{-1}	As(III) +UV	C_0=0.91 mg L^{-1}<br>t_e=2 h	0-55 mg L^{-1}	T=27 °C<br>pH=7<br>dose=0.32 g L^{-1}	**Pseudo-second order:**<br>q_e=3.07 mg g^{-1}<br>k_2=0.031 g mg^{-1} min^{-1}<br>R^2=0.994<br>**Langmuir:**<br>q_m=30.96 mg g^{-1}<br>k_L=0.6538 L mg^{-1}<br>R^2=0.997<br>**Removal:**<br>>90 %	[86]
					As(V)	C_0=1.06 mg L^{-1}<br>t_e=2 h	0-55 mg L^{-1}		**Pseudo-second order:**<br>q_e=3.35 mg g^{-1}<br>k_2=0.147 g mg^{-1} min^{-1}<br>R^2=0.999<br>**Langmuir:**<br>q_m=36.36 mg g^{-1}<br>k_L=0.5288 L mg^{-1}<br>R^2=0.995<br>**Removal:**<br>>90 %	
Fe_3O_4		TEM: 50 nm		38.9 emu g^{-1}	As(V)	-	2-75 mg L^{-1}	T=27 °C	**Langmuir:**	[87]

	A one-pot hydrothermal process		30.1 m^2 g^{-1}					pH=7<br>dose=0.1 g L^{-1}	q_m=92.85 mg g^{-1}<br>k_L=0.046 L mg^{-1}<br>R^2=0.90<br>**Freundlich:**<br>k_F=9.10 mg^{1+n} g^{-1} L<br>1/n=0.49<br>R^2=0.96	
Fe_3O_4	Obtained from red mud	TEM: 8-13 nm (9 nm)<br>XRD (SAXS): 9.77 nm<br>spherical shape	-	55.3 emu g^{-1}	As(V)	C_0=50 μg L^{-1}<br>t_e=45 min	10-1000 μg L^{-1}	T=25 °C<br>pH=2.5<br>dose=8 g L^{-1}	**Pseudo-second order:**<br>q_e=5.21 μg g^{-1}<br>k_2=0.08 g mg^{-1} min^{-1}<br>R^2=0.999<br>**Langmuir:**<br>q_m=400 μg g^{-1}<br>k_L=0.003 L $μg^{-1}$<br>R^2=0.968<br>**Removal:**<br>99.2 %	[88]

Table S2: Adsorption parameters for removal of arsenic and other pollutants from water using diverse magnetic nanohybrids.

Adsorbent	Synthesis method	Size and shape	Surface area	Saturation magnetization	Adsorbates	Initial concentration (C_0) and equilibrium time (t_e)	Concentration range in isotherm experiments	Adsorption conditions	Other pollutants	Kinetic and isothermal parameters	Ref.
Fe_3O_4	Co-precipitation	TEM: 10 nm spherical NPs	-	-	As(III)	C_0=10 µmol L^{-1}<br>t_e=24 h	-	T=27 °C<br>pH=6-8<br>dose=1 g L^{-1}	HA<br>5 mg L^{-1}	Removal=90%	[90]
						C_0=100 µmol L^{-1}<br>t_e=24 h			HA<br>5 mg L^{-1}	Removal=94.3%	
						C_0=10 µmol L^{-1}<br>t_e=24 h			HA<br>50 mg L^{-1}	Removal=90.1%	
						C_0=100 µmol L^{-1}<br>t_e=24 h			HA<br>50 mg L^{-1}	Removal=89.3%	
GO-Fe_3O_4-HA	Co-precipitation	TEM: Fe_3O_4 (8 nm) assembled along the GO sheets.	0.9060 m^2 g^{-1}	-	As(III)	C_0=10 mg L^{-1}<br>t_e=24 h	1 - 10 mg L^{-1}	T=23 °C<br>pH=7<br>dose=0.2 g L^{-1}	HA<br>2 g L^{-1}	**Langmuir:**<br>q_m=16 mg g^{-1}<br>k_L=0.787 L mg^{-1}	[91]
					As(V)	C_0=10 mg L^{-1}<br>t_e=24 h	1 - 10 mg L^{-1}	T=23 °C<br>pH=7<br>dose=0.2 g L^{-1}	HA<br>2 g L^{-1}	**Langmuir:**<br>q_m=16 mg g^{-1}<br>k_L=0.787 L mg^{-1}	
HA-Fe_3O_4	Co-precipitation	TEM: 10.6 nm	-	68.1 emu g^{-1}	As(III)<br>As(V)	C_0=0.2 mg L^{-1}<br>As(III)<br>C_0=0.2 mg L^{-1}<br>As(V)<br>t_e= 3 h	1 - 10 mg L^{-1}	T=25 °C<br>pH=6.6<br>dose=0.2 g L^{-1}	-	**Pseudo-second order:**<br>q_e=0.96 mg g^{-1} As(III)<br>k_2=0.35 g mg^{-1} min^{-1}<br>R^2=0.99<br>q_e =0.99 mg g^{-1} As(V)<br>k_2=1.31 g mg^{-1} min^{-1}<br>R^2=0.99<br>**Langmuir:**<br>q_m=12.2 mg g^{-1} As(III)<br>k_L=3.46 L mg^{-1}<br>R^2=0.96<br>q_m=12.6 mg g^{-1} As(V)<br>k_L=4.88 L mg^{-1}<br>R^2=0.94<br>**Freundlich:**<br>k_F=2.96 L g^{-1} As(III)<br>n=1.75<br>R^2=0.99<br>k_F=2.30 L g^{-1} As(V)<br>n=3.57<br>R^2=0.99	[92]

						C_0=0.2 mg L^{-1} As(III) C_0=0.2 mg L^{-1} As(V) t_e= 3 h	-	T=25 °C pH=6.6 dose=0.2 g L^{-1}	Fe^{2+} (1.0 mg L^{-1})	Removal=97% As(III) Removal=98% As(V)	
							-	T=25 °C pH=6.6 dose=0.2 g L^{-1}	Cl^- (1.0 mmol L^{-1}) NO_3^- (1.0 mmol L^{-1}) SO_4^{2-}(1.0 mmol L^{-1})	Removal>95% As(III) Removal>98% As(V)	
							-	T=25 °C pH=6.6 dose=0.2 g L^{-1}	PO_4^{3-}(1.0 mmol L^{-1})	Removal<85% As(III) Removal<95% As(V)	
							-	T=25 °C pH=6.6 dose=0.2 g L^{-1}	CO_3^{2-}(1.0 mmol L^{-1})	Removal>95% As(III) Removal<30% As(V)	
NC-MA /L-Fe_3O_4	Magnetic stirring and ultrasonic treatment	XRD: 22 nm Fe_3O_4<br>TEM: 22 nm of grain sizes of Fe_3O_4 present on NC-MA /L surface<br>Pore diameter: 15.2 nm<br>Pore volume: 0.71 cm^3 g^{-1}	85.3 m^2 g^{-1}	-	As(V)	C_0=10 mg L^{-1} t_e= 90 min	0.1 - 20 mg L^{-1}	T=25 °C pH=6 dose=0.1 g L^{-1}	**-**	**Pseudo-second order:** q_e=1.0463 mg g^{-1} k_2=0.189 g mg^{-1} min^{-1} R^2=0.997<br>**Freundlich:** k_F=0.2412 mol g^{-1} (L mol^{-1})$^{1/n}$ n=1.779 R^2=0.995<br>**Adsorption capacities:** 85.3 mg g^{-1}	[93]
					As(V)	C_0=10 mg L^{-1} t_e= 90 min	-	T=25 °C pH=6 dose=0.1 g L^{-1}	PO_4^{3-}(5.6 mg L^{-1}) SO_4^{2-}(42.5 mg L^{-1}) Ca^{2+} (15.3 mg L^{-1}) Mg^{2+} (9.1 mg L^{-1}) SO_4^{4-}(5 mg L^{-1}) Cl^- (1.2 mg L^{-1}) HCO_3^- (856 mg L^{-1})	Adsorption capacities: 69.1 mg g^{-1}	
Fe_3O_4	Reduction-precipitation method	TEM: 25 nm spherical NPs	-	-	As(V)	C_0=13 mg L^{-1} t_e= 1 h	-	T=25 °C pH=2.5 dose=1 g L^{-1}	-	**Pseudo-second order:** q_e=5.94 mg g^{-1}	[94]

Produced from iron ore tailings									k_2=0.039 g mg^{-1} min^{-1}<br>R^2=0.99<br>**Langmuir:**<br>q_m=9.72 mg g^{-1}<br>k_L=1.17 L mg^{-1}<br>R^2=0.996<br>**maximum adsorption capacity:**<br>78.95 %<br>9.72 mg/g<br>Contact time=6 h	
				As(V)	C_0=13 mg L^{-1}<br>t_e= 1 h	-	T=25 °C<br>pH=5.5<br>dose=2 g L^{-1}	Cu^{2+} (13 mg L^{-1})<br>Zn^{2+} (13 mg L^{-1})<br>Mn^{2+} (13 mg L^{-1})<br>Simultaneous co-adsorption	**Pseudo-second order:**<br>q_e=5.97 mg g^{-1}<br>k_2=0.070 g mg^{-1} min^{-1}<br>R^2=0.99<br>**Langmuir:**<br>q_m=9.79 mg g^{-1}<br>k_L=2.30 L mg^{-1}<br>R^2=0.999<br>**Maximum adsorption capacity:** 9.79 mg g^{-1}<br>Adsorption order of metals can be arranged as:<br>As^{5+}>Cu^{2+}>Zn^{2+}> Mn^{2+}<br>Contact time=6 h	
				As(V)	C_0=13.2 mg L^{-1}<br>t_e= 1 h	-	T=25 °C<br>pH=2.5<br>dose=1 g L^{-1}	Al^{3+} (13 mg L^{-1})	Removal=72.37 % As(V)	
				As(V)	C_0=13.4 mg L^{-1}<br>t_e= 1 h	-	T=25 °C<br>pH=2.5<br>dose=1 g L^{-1}	Al^{3+} (103 mg L^{-1})	Removal=68.14 % As(V)	
				As(V)	C_0=13.3 mg L^{-1}<br>t_e= 1 h	-	T=25 °C<br>pH=2.5<br>dose=1 g L^{-1}	Fe^{3+}(13.4 mg L^{-1})	Removal=70.83 % As(V)	
				As(V)	C_0=13.4 mg L^{-1}<br>t_e= 1 h	-	T=25 °C<br>pH=2.5<br>dose=1 g L^{-1}	Fe^{3+}(104.5 mg L^{-1})	Removal=65.25 % As(V)	
Co-precipitation with sodium	XRD: 8 nm<br>Pore radius: 5.5 nm<br>Pore volume: 0.29 cm^3 g^{-1}	137.4 m^2 g^{-1}	65.8 emu g^{-1}	As(III)<br>Pb(II)<br>Cd(II)	C_0=20 g L^{-1}<br>t_e= 20 min	20–150 mg L^{-1}	T=27 °C<br>pH=5.5<br>dose=5 g L^{-1-}	-	**Pseudo-second order:**<br>q_e=0.43 mg g^{-1} As(III)<br>k_2=1.56 g mg^{-1} min^{-1}<br>R^2=0.9997	[95]

Fe_3O_4	hydroxide using an aqueous extract lemon peel as surfactant (green Fe_3O_4 nanoparticles)				simultaneous					q_e=0.88 mg g^{-1} Pb(II)<br>k_2=2.24 g mg^{-1} min^{-1}<br>R^2=0.9999<br>q_e=0.42 mg g^{-1} Cd(II)<br>k_2=0.54 g mg^{-1} min^{-1}<br>R^2=0.9923<br>**Langmuir:**<br>q_m=25.91 mg g^{-1} As(III)<br>k_L=0.07 L mg^{-1}<br>R^2=0.9905<br>q_m=9.01 mg g^{-1} Pb(II)<br>k_L=0.01 L mg^{-1}<br>R^2=0.9932<br>q_m=25.84 mg g^{-1} Cd(II)<br>k_L=0.08 L mg^{-1}<br>R^2=0.9949<br>**Freundlich:**<br>k_F=0.24 L mg^{-1} As(III)<br>n=3.31<br>R^2=0.9861<br>k_F=2.10 L mg^{-1} Pb(II)<br>n=1.58<br>R^2=0.988<br>k_F=0.29 L mg^{-1} Cd(II)<br>n=5.60<br>R^2=0.8756<br>**Adsorption capacities:**<br>98.8 Pb(II)<br>46.0 Cd(II)<br>48.2 As(III)<br>Contact time=2 h	
					As(III)<br>Pb(II)<br>Cd(II)<br>simultaneous	C_0(As(III))=129 g L^{-1}<br>C_0(Pb(II))= 0.9 g L^{-1}<br>C_0(Pb(II))= 0.9 g L^{-1}<br>t_e= 20 min	-	T=27 °C<br>pH=5.5<br>dose=5 g L^{-1-}	Cl^- (28.6 mg L^{-1})<br>NO_3^- (1.5 mg L^{-1})<br>PO_4^{3-}(3 mg L^{-1})<br>SO_4^{2-}(3.5 mg L^{-1})<br>Ca^{2+}(49.1 mg L^{-1})<br>Mg^{2+}(16.6 mg L^{-1}) Na^+(105 mg L^{-1}) K^+(1.04 mg L^{-1})	Removal>95% As(III)<br>Removal<5% Pb(II)<br>Removal<2.5% Cd(II)	
Fe_3O_4/nOG	Obtained by physical exfoliation method using thermal treatment at	TEM: 5-9 nm Fe_3O_4	189.94 m^2 g^{-1}	-	As(III)<br>As(V)	C_0(As(III))=1 mg L^{-1}<br>C_0(As(V))=1 mg L^{-1}	-	T=25 °C<br>pH=7<br>dose=0.1 g L^{-1-}		**Pseudo-second order:**<br>q_e=5.18 mg g^{-1} As(III)<br>k_2=0.0031 g mg^{-1} min^{-1}<br>R^2=0.998<br>q_e=3.84 mg g^{-1} As(V)<br>k_2=0.0098 g mg^{-1} min^{-1}<br>R^2=0.999 **Langmuir:**	[96]

600 °C for 1 h					-	q_m=24.6 mg g^{-1} As(III)<br>k_L=0.6 L mg^{-1}<br>R^2=0.856<br>q_m=9.44 mg g^{-1} As(V)<br>k_L=0.78 L mg^{-1}<br>R^2=0.767<br>**Freundlich:**<br>k_F=7.49 mg g^{-1} (L mg^{-1})$^{1/n}$ As(III)<br>n=3.45<br>R^2=0.974 k_F=3.82 mg g^{-1} (L mg^{-1})$^{1/n}$ As(V)<br>n=4.76<br>R^2=0.981<br>**Maximum adsorption capacity:**<br>38 mg L^{-1} As(III)<br>14 mg L^{-1} As(V)
	As(III)<br>As(V)	C_0(As(III))=5 mg L^{-1}<br>C_0(As(V))=5 mg L^{-1}<br>t_e= 24 h	-	T=25 °C<br>pH=7<br>dose=0.1 g L^{-1}	-	**Adsorption capacities:** 11.3 mg g^{-1} As(III)<br>5.5 mg g^{-1} As(V)<br>control without competitive anion
	As(III)<br>As(V)	C_0(As(III))=5 mg L^{-1}<br>C_0(As(V))=5 mg L^{-1}<br>t_e= 24 h	-	T=25 °C<br>pH=7<br>dose=0.1 g L^{-1}	Cl^- (1 mmol L^{-1})<br>NO_3^- (1 mmol L^{-1}) HCO_3^-(1 mmol L^{-1})	Adsorption capacities<11 mg g^{-1} As(III)<br>Adsorption capacities<5.5 mg g^{-1} As(V)<br>94-98% compared to the control
	As(III)<br>As(V)	C_0(As(III))=5 mg L^{-1}<br>C_0(As(V))=5 mg L^{-1}<br>t_e= 24 h	-	T=27 °C<br>pH=7<br>dose=0.1 g L^{-1}	SO_4^{2-}(1 mmol L^{-1})	Adsorption capacities<8 mg g^{-1} As(III)<br>Adsorption capacities<4 mg g^{-1} As(V)<br>65–72% compared to the control
	As(III)<br>As(V)	C_0(As(III))=5 mg L^{-1}<br>C_0(As(V))=5 mg L^{-1}<br>t_e= 24 h	-	T=25 °C<br>pH=7<br>dose=0.1 g L^{-1}	PO_4^{3-}(1 mmol L^{-1})	Adsorption capacities<3.8 mg g^{-1} As(III)<br>Adsorption capacities<3 mg g^{-1} As(V)<br><br>31% As(III)<br>52% As(V)<br>compared to the control

					As(III)<br>As(V)	C_0(As(III))=5 mg L^{-1}<br>C_0(As(V))=5 mg L^{-1}<br>t_e= 24 h	-	T=25 °C<br>pH=7<br>dose=0.1 g L^{-1-}	HA (1 mg L^{-1})	Adsorption capacities=8 mg g^{-1} As(III)<br>Adsorption capacities=5 mg g^{-1} As(V)	
Fe_3O_4@sand	Coprecipitation	FESEM: 20-60 nm size of Fe_3O_4 NPs	-	-	As(V)	C_0=100 mg L^{-1}<br>t_e= 330 min	1 – 15.35 mg L^{-1}	T=30 °C<br>pH=2-7<br>dose=1 g L^{-1-}	Cr^{3+}(100 mg L^{-1})<br>Mg^{2+}(100 mg L^{-1})<br>Ni^{2+}(100 mg L^{-1})<br>Pb^{2+}(100 mg L^{-1})<br>Cd^{2+}(100 mg L^{-1})<br>Zn^{2+}(100 mg L^{-1})<br>Fe^{3+}(100 mg L^{-1})	**Pseudo-second order:**<br>q_e=0.0406 mg g^{-1}<br>k_2=3.754 g mg^{-1} min^{-1}<br>R^2=0.990 **Langmuir:**<br>q_m=0.639 mg g^{-1}<br>k_L=216.99 L mg^{-1}<br>R^2=0.993<br>**Freundlich:**<br>k_F=2.065 mg g^{-1}<br>n=2.39<br>R^2=0.984<br>**Adsorption capacities:** 2.06 mg g^{-1} As(V)<br>94%< As(V)<99%<br>Effect removal efficiency Cr^{3+}> Mg^{2+}> Ni^{2+}> Pb^{2+}> Cd^{2+}>Zn^{2+} >Fe^{3+}<br>Contact time: 420 min	[97]
PAN/GO/γ-Fe_2O_3	Electrospinning technique	TEM: 6-10 nm γ-Fe_2O_3<br><br>PAN diameter: 250-400 nm	30.24 m^2 g^{-1}	3.90 emu g^{-1}	As(V)	C_0=5 mg L^{-1}<br>t_e= 90 min	1 - 50 mg L^{-1}	T=30 °C<br>pH=4<br>dose=0.6 g L^{-1}	-	**Pseudo-second order:**<br>q_e=8.904 mg g^{-1}<br>k_2=0.006 g mg^{-1} min^{-1}<br>R^2=0.9933<br>**Langmuir:**<br>q_m=36.1 mg g^{-1}<br>k_L=0.62 L g^{-1}<br>R^2=0.9947<br>**Freundlich:**<br>k_F=2.76 mg g^{-1}<br>n=2.27<br>R^2=0.9393<br>**Adsorption capacities:** $q_{e,exp}$=7.95 mg g^{-1}<br>95.72%<br>Contact time: 2 h<br>**Maximum adsorption capacity:**<br>36.1 mg g^{-1}	[98]

	As(V)	C_0=5 mg L^{-1}<br>t_e= 90 min	-	T=30 °C<br>pH=4<br>dose=0.6 g L^{-1}	Cl^-<br>NO_3^-<br>SO_4^{2-}	Adsorption capacities=7.95 mg g^{-1}
	As(V)	C_0=5 mg L^{-1}<br>t_e= 90 min	-	T=30 °C<br>pH=4<br>dose=0.6 g L^{-1-}	PO_4^{3-}	Adsorption capacities< 5 mg g^{-1}

Table S3. Lead, other metals, and pollutants adsorption parameters for magnetic nanohybrids.

Adsorbent	Synthesis method	Size and shape	Surface area	Saturation magnetization	Adsorbates	Initial concentration (C_0) and equilibrium time (t_e)	Concentration range in isotherm experiments	Adsorption conditions	Kinetic and isothermal parameters	Ref.
$\gamma-Fe_2O_3-1$	Co-precipitation	TEM: 4 - 10nm<br>XRD: 6.4 nm	77.5-214 m² g^{-1}	12 – 62 emu g^{-1}	Pb(II)<br>Cu(II)	C_0=50 mg L^{-1} t_e=7 h<br>C_0=40 mg L^{-1} t_e=7 h	0–50 mg L^{-1}	T=25 °C<br>pH=7<br>dose=0.56 g L^{-1}	**Langmuir:**<br>q_m=57.3-88.2 mg g^{-1}	[43]
$\gamma-Fe_2O_3-2$		XRD: 7.1 nm								
$\gamma-Fe_2O_3@SiO_2$		XRD: 10 nm								
$\gamma-Fe_2O_3-SBA15$		XRD: 6.2 nm								
$\gamma-Fe_2O_3@OA$		XRD: 5.9 nm								
$\gamma-Fe_2O_3@LA$		XRD: 4.5 nm								
$\gamma-Fe_2O_3@L-arg$		XRD: 5.9 nm								
$\gamma-Fe_2O_3@HAp$		XRD: 8.0 nm								
$\gamma-Fe_2O_3-EDTA1$		XRD: 3.0 nm								
$\gamma-Fe_2O_3@MWCNTs$		XRD: 7.3 nm								

Fe_3O_4	Co-precipitation	TEM: 8-13 nm-spherical	-	-	Pb(II)	C_0=50 mg L^{-1} t_e=25 min	10-50 mg L^{-1}	T=25 °C<br>pH=5.0<br>dose=0.02 g L^{-1}	**Pseudo- First order:**<br>k_1=0.053 g mg^{-1} h^{-1}<br>R^2=0.0184<br>**Pseudo Second order:**<br>k_2=0.05 g mg^{-1} h^{-1}<br>R^2=0.99387	[106]
$\gamma - Fe_2 0_3 - EDTA1$<br>$\gamma - Fe_2 0_3 - EDTA2$<br>$\gamma - Fe_2 0_3 - EDTA3$	Co-precipitation	TEM: 4 nm $\gamma - Fe_2 0_3 -$ EDTA1<br>TEM: ~7.6 nm $\gamma - Fe_2 0_3 - EDTA2$<br>TEM: ~7 nm $\gamma - Fe_2 0_3 - EDTA2$	272 $m^2 g^{-1}$	16.3 emu g^{-1}	Pb(II)	C_0=50 mg L^{-1}<br>t_e=7 h	0–50 mg L^{-1}	T=70 °C, 80 °C<br>pH=5.5<br>dose=0.5 g L^{-1}	-	[30]
Fe_3O_4	Co-precipitation	TEM: 8-13 nm	12.7 $m^2 g^{-1}$	65.3-75.8 emu g^{-1}	Pb(II)<br><br>Cr(II)	C_0=25,50,100 mg L^{-1} t_e=3 h	-	T=25 °C, 35 °C, 45 °C, pH=5.0<br>dose=0.2 g L^{-1}<br>T=25 °C, 35 °C, 45 °C,<br>pH=2.0,<br>dose=0.1 g L^{-1}	**Langmuir:**<br>q_m=52.9 mg g^{-1} (25 °C)<br>q_m=52.8 mg g^{-1} (35 °C)<br>q_m=53.2 mg g^{-1} (45 °C)<br>R^2 =0.955-0.981<br>**Langmuir:**<br>q_m=20.2 mg g^{-1} (25 °C)<br>q_m=26.8 mg g^{-1} (35 °C)<br>q_m=34.9 mg g^{-1} (45 °C)	[107]
GFMNPECABs	Co-precipitation	-	-	1.7 emu g^{-1}	Pb(II)	C_0=10-80 mg L^{-1}<br>t_e=100 min	10-80 mg L^{-1}	T=40 °C,<br>50 °C, 60 °C,<br>pH=6.0,<br>dose=10 g L^{-1}	**Langmuir:**<br>q_m=473.9 mg g^{-1} (40 °C)<br>R^2=0.993 (40 °C)<br>k_L=0.0011 L mg^{-1} (40 °C)<br>q_m =555.5 mg g^{-1} (50 °C)<br>R^2=0.998 (50 °C)<br>k_L=0.0016 L mg^{-1} (50 °C)<br>q_m =555.5 mg g^{-1} (60 °C)<br>R^2=0.998 (60 °C)<br>k_L=0.0016 L mg^{-1} (60 °C)<br>**Freundlich:**<br>k_F=2.139 L mg^{-1} (40 °C)<br>R^2=0.953 (40 °C)<br>k_F=2.280 L mg^{-1} (50 °C)<br>R^2=0.968 (50 °C)	[108]

									k_F=2.917 L mg^{-1} (60 °C)<br>R^2=0.973 (60 °C)<br><br>**Temkin:**<br>K_t=5.150 L mg^{-1} (40 °C)<br>R^2=0.919 (40 °C)<br><br>K_t=5.340 L m^{-1} g^{-1} (50 °C)<br>R^2=0.940 (50 °C)<br><br>K_t=10.39 L mg^{-1} (60 °C)<br>R^2=0.940 (60 °C)	
					Pb(II)			T=25 °C,<br>35 °C, 45 °C,<br><br>pH=6.0,<br>dose=2.0 g L^{-1}	**Langmuir:**<br>q_e=18.8 mg g^{-1}<br>q_m=7.00 mg g^{-1} (25 °C)<br>q_m=11.6 mg g^{-1} (35 °C)<br>q_m=18.8 mg g^{-1} (45 °C)	
L-Cyst-Fe_3O_4	Co-precipitation	XRD: 15 nm	-	-	Cr(VI)	C_0=50 mg L^{-1} t_e=1 h	10-100 mg L^{-1}	T=25 °C,,<br>35 °C, 45 °C,<br><br>dose=2.0 g L^{-1},<br>pH=2.0	**Langmuir:**<br>q_e=34.5 mg g^{-1}<br>q_m=11.6 mg g^{-1} (25 °C)<br>q_m=23.5 mg g^{-1} (35 °C)<br>q_m=34.5 mg g^{-1} (45 °C)	[109]
					Pb(II)			T=25 °C,<br>35 °C, 45 °C,<br><br>pH=6.0,<br>dose=2.0 g L^{-1}	**Freundlich:**<br>k_F=19.16 mg g^{-1} (25 °C)<br>k_F=23.88 mg g^{-1} (35 °C)<br>k_F=31.19 mg g^{-1} (45 °C)	
					Cr(VI)			T=25 °C,<br>35 °C, 45 °C,<br>pH=2.0,<br>dose=2.0 g L^{-1}	**Freundlich:**<br>k_F=25.98 mg g^{-1} (25 °C)<br>k_F=30.27 mg g^{-1} (35 °C)<br>k_F=41.05 mg g^{-1} (45 °C)	
					Pb(II)				**Langmuir:**<br>q_m=257 mg g^{-1}	
Fe_3O_4-NH_2	Solvent thermal	TEM: 100 nm	-	80 emu g^{-1}		C_0=25, 35, 45 mg L^{-1}	30-300 mg L^{-1}	T=25 °C,<br>35 °C, 45 °C,<br>pH=5.5		[110]
					Cd(II)				**Langmuir:**<br>q_m=129.5 mg g^{-1}	

Fe_3O_4	-	TEM: 260 nm	-	69.4 emu g^{-1}	C_0=10 mg L^{-1} t_e=3 h	Pb(II)	0-80 mg L^{-1}	T=25 °C, 40 °C, 55 °C, pH=5.0, dose=0.2 g L^{-1}	**Langmuir:** q_e=41.04 mg g^{-1} (25 °C) q_e=46.69 mg g^{-1} (40 °C) q_e= 55.84 mg g^{-1} (55 °C) **Freundlich:** k_F=18.28 $mL^{1/n}$ $\mu g^{1-1/n}$ (25 °C) k_F=19.44 $mL^{1/n}$ $\mu g^{1-1/n}$ (40 °C) k_F=20.58 $mL^{1/n}$ $\mu g^{1-1/n}$ (55 °C)	[111]
						Cu(II)	0-70 mg L^{-1}	T=25 °C, 40 °C, 55 °C, pH=6.0, dose=0.2 g L^{-1}	**Langmuir:** q_e=21.14 mg g^{-1} (25 °C) q_e=31.92 mg g^{-1} (40 °C) q_e=42.85 mg g^{-1} (55 °C) **Freundlich:** k_F=2.32 $mL^{1/n}$ $\mu g^{1-1/n}$ (25 °C) k_F=3.65 $mL^{1/n}$ $\mu g^{1-1/n}$ (40 °C) k_F=4.16 $mL^{1/n}$ $\mu g^{1-1/n}$ (55 °C)	
Fe_3O_4@PDA	-	TEM: 260-300 nm	-	49 emu g^{-1}	C_0=10 mg L^{-1} t_e=3 h	Pb(II)	0-70 mg L^{-1}	T=25 °C, 40 °C, 55 °C, pH=5.0, dose=0.2 g L^{-1}	**Langmuir:** q =57.25 mg g^{-1} (25 °C) q_e=71.08 mg g^{-1} (40 °C) q_e=101.96 mg g^{-1} (55 °C) **Freundlich:** k_F=28.43 $mL^{1/n}$ $\mu g^{1-1/n}$ (25 °C) k_F=36.19 $mL^{1/n}$ $\mu g^{1-1/n}$ (40 °C) k_F=41.96 $mL^{1/n}$ $\mu g^{1-1/n}$ (55 °C)	[111]
						Cu(II)	0-70 mg L^{-1}	T=25 °C, 40 °C, 55°C, pH=6.0, dose=0.2 g L^{-1}	**Langmuir:** q_e=86.35 mg g^{-1} (25 °C) q_e=104.81 mg g^{-1} (40 °C) q_e=112.48 mg g^{-1} (55 °C) **Freundlich:** k_F= 22.35 $mL^{1/n}$ $\mu g^{1-1/n}$ (25 °C) k_F=24.73 $mL^{1/n}$ $\mu g^{1-1/n}$ (40 °C) k_F=30.88 $mL^{1/n}$ $\mu g^{1-1/n}$ (55 °C)	

Fe_3O_4	-	-	-	-	Pb(II)<br><br>Cd(II)	C_0=100 mg L^{-1}<br>t_e=20 min	50-200 mg L^{-1}	T=25 °C,<br>pH=5.5,<br>dose=5.0 g L^{-1}	**Langmuir:**<br>q_m=13.88 mg g^{-1} Pb(II)<br>q_m=9.52 mg g^{-1} Cd(II)<br>**Freundlich**<br>k_F=2.99 L g^{-1} Pb(II)<br>k_F =3.46 L g^{-1} Cd(II)<br>**Dubinbin–Radushkevich:**<br>q_{mDR}=12.88 mg g^{-1} Pb(II)<br>q_{mDR}=10.07 mg g^{-1} Cd(II)<br>**Pseudo- First order:**<br>q_e=3.45 mg g^{-1} Pb(II)<br>k_1=0.274 g mg^{-1} h^{-1}<br>q_e=6.48 mg g^{-1} Cd(II)<br>k_1=0.213 g mg^{-1} h^{-1}<br><br>**Pseudo Second order:**<br>q_e=11.11 mg g^{-1} Pb(II)<br>k_2=0.090 g mg^{-1} h^{-1}<br>q_e=10.6 mg g^{-1} Cd(II)<br>k_2=0.214 g mg^{-1} h^{-1}	[112]
Fe_3O_4-ETT	Via epoxide ring opening reaction	FESEM: 25-52 nm<br>TEM: spherical, mesoporous NPs	84.8 m^2 g^{-1}	30.7 emu g^{-1}	Pb(II)	C_0=50 mg L^{-1}<br>t_e=20 min	-	T=19.85 °C,<br>pH=5,<br>dose=5 g L^{-1}	**Pseudo second order:**<br>q_e= 28.98 mg g^{-1}<br>k_2= 0.047 g mg^{-1} min^{-1}<br>R^2 =0.9985<br>**Langmuir:**<br>q_m= 21.05 mg g^{-1}<br>k_L= 0.675 L mg^{-1}<br>R^2 = 0.9729<br>**Freundlich:**<br>k_F= 28.98<br>n=7.25<br>R^2= 0.9911	[113]
Fe_3O_4-PEI/β-CD	solvothermal method	TEM: spherical particle	17.5 m^2 g^{-1}	60.3 emu g^{-1}	Pb(II)	C_0=100 mg L^{-1}<br>t_e=200 min	10 to 100 mg L^{-1}	T=30 °C<br>pH=6<br>dose=0.5 g L^{-1}	**Experimental:**<br>$q_{e,\,exp}$= 39.7 mg g^{-1}<br>**Pseudo Second order:**<br>q_e= 40.0 mg g^{-1}<br>k_2= 0.0009 g mg^{-1} h^{-1}<br>R^2=0.924<br>**Langmuir:**<br>q_m= 73.1 mg g^{-1}<br>k_L= 0.021 L mg^{-1}<br>R^2=0.98<br>**Freundlich:**<br>k_F=3.6<br>n=1.7<br>R^2=0.97	[114]

					methyl orange (MO)	C_0=100 mg L^{-1}<br>t_e=100 min	25-200 mg L^{-1}	T=30 °C<br>pH=3<br>dose=0.5 g L^{-1}	**Pseudo Second order:**<br>q_e=131.1 mg g^{-1}<br>k_2=0.0018 g mg^{-1} h^{-1}<br>R^2=0.977<br>**Langmuir:**<br>q_m=192.2 mg g^{-1}<br>k_L=0.178 L mg^{-1}<br>R^2=0.97	
					methyl orange (MO) Pb(II)	C_0(MO)=100 mg L^{-1}<br>C_0(Pb)=50 mg L^{-1}	Pb(II) 10-50<br>MO 30 and 60 mg L^{-1}	T=30 °C<br>pH=6	MO removal efficiencies=95.4 %<br>Pb(II) removal efficiencies=82.9 %<br>Maximum adsorption capacity Pb(II)=285.3 mg g^{-1}<br>Maximum adsorption capacity Cd(II)=48.2 mg g^{-1}	
$Fe_3O_4@SiO_2@$PEI-NTDA	Fe_3O_4<br>(Solvothermal method)	TEM: 200 -300 nm<br>SEM: spherical particles	-	21.6 emu g^{-1}	Pb(II)	C_0=200 mg L^{-1}<br>t_e=200 min	-	T=25 °C<br>pH=6<br>dose=0.5 g L^{-1}	**Pseudo-second order:**<br>q_e=277.7 mg g^{-1}<br>k_2=0.00360 g mg^{-1} min^{-1}<br>R^2=0.996<br>**Langmuir:**<br>q_m=286.9 mg g^{-1}<br>k_L=0.0786 L mg^{-1}<br>R^2=0.999<br>**Freundlich:**<br>k_F=74.77 mg g^{-1}<br>n=4.20<br>R^2=0.756	[115]
Fe_3O_4-g- C_3N_4	Ultrasonic method<br>Fe_3O_4<br>(precipitation)	SEM: 500 nm-2μm lamellar structure	-	8.4 emu g^{-1}	Pb(II)	C_0=200 mg L^{-1}<br>t_e=20 min	40-240 mg L^{-1}	T=25 °C<br>pH=6<br>dose=1 g L^{-1}	**Pseudo-second order:**<br>q_e=103.47 mg g^{-1}<br>k_2=0.0068 g mg^{-1} min^{-1}<br>R^2= 0.9996<br>**Langmuir:**<br>q_m=189.36 mg g^{-1}<br>k_L=0.0059 L mg^{-1}<br>R^2=0.998	[116]

Fe_3O_4-SO_3H	The modified Stöber sol−gel process	SEM: 80 nm	18.3 $m^2\ g^{-1}$	69 emu g^{-1}	Pb(II)	C_0=10 mg L^{-1}<br>t_e=12 h	-	pH=7<br>dose=1 g L^{-1}	**Freundlich:**<br>k_F=3.66 mg g^{-1}<br>n=1.57<br>R^2= 0.982<br>maximum adsorption capacity: 80.9 mg g^{-1}<br>**Pseudo-second order:**<br>q_e=9.69 mg g^{-1}<br>K_2=0.095 g mg^{-1} min^{-1}<br>R^2=1<br>**Langmuir:**<br>q_m=108.93 mg g^{-1}<br>k_L= 0.373 L mg^{-1}<br>R^2 =0.998	[117]
Fe_3O_4@APS<br>Fe_3O_4@ APS@AA-co-CA	Coprecipitation method	TEM: 15-20 nm	-	79 emu g^{-1}<br>67 emu g^{-1}<br>52 emu g^{-1}	Pb(II)	C_0=100 mg L^{-1}<br><br>t_e=45 min	-	pH=5.5<br>dose=0.05 g L^{-1}	Adsorption capacities:166.1 mg g^{-1}<br>**Second order:**<br>q_e=78.8 mg g^{-1}<br>K_2=0.0057 g mg^{-1} min<br>R^2=0.9968<br>**Langmuir:**<br>q_m=166.1 mg g^{-1}<br>k_L=0.1379 L mg^{-1}<br>R^2=0.9992	[118]
CNTs/ Fe_3O_4<br>MPTS-CNTs/ Fe_3O_4	Thermal decomposition	TEM: 6 nm	88.4 $m^2\ g^{-1}$<br>97.2 $m^2\ g^{-1}$	22.9 emu g^{-1}<br>29 emu g^{-1}	Pb(II)	C_0=50 mg L^{-1}	5–90 mg L^{-1}	pH=6.5<br>dose=1 g L^{-1}	maximum adsorption capacity: 65.40 mg g^{-1}<br>**Pseudo-second order**<br>q_e=48.28 mg g^{-1}<br>K_2=0.0131 g mg^{-1} min<br>R^2=0.9826<br>**Langmuir**<br>q_m=65.40 mg g^{-1}<br>k_L=0.0453 L mg^{-1}<br>R^2=0.9935	[119]
Fe_3O_4@C@TiO_2<br>(0FeCTi)	Oil - phase cyclic magnetic adsorption (OCMA) method	BET: pore size adsorption=19.34 nm desorption=16.56 nm nanotubes	37 $m^2\ g^{-1}$	-	Pb(II)<br>Rhodamine B (RhB) simultaneous	C_0=100 mL mixed polluted water containing<br>2 mg Pb(II)<br>2 mg Rhb<br>t_e=3 h	-	T=25 °C<br>pH=7<br>dose=1 g L^{-1}	**Pseudo-first order model:**<br>**Pb(II)**<br>k_1=0.048 min^{-1}<br>R^2=0.9874	[120]
Fe_3O_4@C@TiO_2<br>(1FeCTi)	Oil - phase cyclic magnetic adsorption (OCMA) method	BET: pore size adsorption=16.66 nm desorption=13.46 nm nanotubes	41.7 $m^2\ g^{-1}$	2.1 emu g^{-1}	Pb(II)<br>Rhodamine B (RhB) simultaneous	C_0=100 mL mixed polluted water containing<br>2 mg Pb(II)<br>2 mg Rhb<br>t_e=3 h	-	T=25 °C<br>pH=7<br>dose=1 g L^{-1}	Removal rate: 98% RhB, 92% Pb<br>**Pseudo-first order model:**<br>**Pb(II)**<br>k_1=0.027 min^{-1}<br>R^2=0.9991<br>**Adsorption--reaction model:**	

Fe_3O_4@C@TiO_2 (3FeCTi)	Oil - phase cyclic magnetic adsorption (OCMA) method	BET: pore size<br>adsorption=16.08 nm<br>desorption=8.66 nm<br>nanotubes	50.3 $m^2\ g^{-1}$	3.9 emu g^{-1}	Pb(II)<br>Rhodamine B (RhB)<br>simultaneous	C_0=100 mL mixed polluted water containing<br>2 mg Pb(II)<br>2 mg Rhb<br>t_e=3 h	-	T=25 °C<br>pH=7<br>dose=1 g L^{-1}	**Pb(II)**<br>R^2=0.9766<br>**Pseudo-first order model:**<br>**Pb(II)**<br>k_1=0.023 min^{-1}<br>R^2=0.9590<br>**Adsorption-reaction model:**<br>**Pb(II)**<br>R^2=0.9980	
Fe_3O_4 -$FeMoS_4$-MgAl-LDH	precipitation	FE-SEM: 35 nm<br>SEM: layered morphology with often hexagonal crystal shape	-	60 emu g^{-1}	Pb(II)<br>Cd(II)<br>Cu(II)<br>simultaneous	C_0(Pb(II))=20 mg L^{-1}<br>C_0(Cd(II))=20 mg L^{-1}<br>C_0(Cu(II))=20 mg L^{-1}<br>t_e=60 min	10-300 mg L^{-1}	T=25 °C<br>pH=5<br>dose=1.5 g L^{-1}	sonication time: 20 min<br>**For Pb(II)**<br>**Pseudo-second order:**<br>q_e=114.84 mg g^{-1}<br>k_2= 0.0027 min^{-1}<br>R^2=0.999<br>**Experimental:**<br>q_e= 190.75 mg g^{-1}<br>**Langmuir:**<br>q_m=188.84 mg g^{-1}<br>k_L =0.0546 L mg^{-1}<br>R^2=0.9861<br>**Freundlich:**<br>k_F=2.363 L mg^{-1}<br>n=1.585<br>R^2=0.9106	[121]
SH-mSi@ Fe_3O_4	Modified Stöber method	SEM and TEM: 500 nm	321 $m^2\ g^{-1}$	38.4 emu g^{-1}	Pb(II)	C_0=0-0.6 mg L^{-1}<br>t_e=20 min	-	dose= 0.01 g L^{-1}<br>pH=6.5	**Pseudo second order:**<br>K_2: 0.430 g $mmol^{-1}$ min<br>**Langmuir:**<br>q_m :0.442 mmol g^{-1}<br>k_L: 22.3 L $mmol^{-1}$<br>R^2=0.999	[122]
mHAP-oMWCNTs	Fe_30_4(Hydrothermal method)	SEM: spindly tubular structure with the average tube size of ~40 nm mHAP-oMWCNTs had the cylinder structure with opens at both ends.	212 $m^2\ g^{-1}$	33 emu g^{-1}	Pb(II)	C_0=100 mg L^{-1}<br>t_e=40 min	40–200 mg L^{-1}	T=25 °C<br>pH=4.1<br>dose=0.1 g L^{-1}	**Pseudo-second order:**<br>q_e = 472.84 mg g^{-1}<br>k_2 = 0.1424 mg min g^{-1}<br>R^2=0.9996<br>**Freundlich:**<br>k_F= 502.2 mg g^{-1}<br>n=2.42<br>R^2= 0.9204<br>**Langmuir:**<br>q_m=681.2 mg g^{-1}<br>k_L=2.599 L mg^{-1}<br>R^2= 0.998	[123]
